# Lipocalin 15 in the olfactory mucus is a biomarker for Bowman’s gland activity

**DOI:** 10.1038/s41598-022-13464-y

**Published:** 2022-06-24

**Authors:** Chiori Ijichi, Kenji Kondo, Masayoshi Kobayashi, Ayaka Shirasawa, Kazutaka Shimbo, Kunio Nakata, Yutaka Maruyama, Yusuke Ihara, Yayoi Kawato, Teruhisa Mannen, Rie Takeshita, Yoshimi Kikuchi, Yuki Saito, Tatsuya Yamasoba

**Affiliations:** 1 Food Products Division, Technology & Solution Development Center, Institute of Food Science and Technologies, Ajinomoto Co., Inc., Kawasaki, 210-8681 Japan; 2 Department of Otorhinolaryngology-Head and Neck Surgery, Graduate School of Medicine and Faculty of Medicine, The University of Tokyo, Tokyo, 113-8655 Japan; 3 Department of Otorhinolaryngology-Head and Neck Surgery, Mie University Graduate School of Medicine, Tsu, Mie Japan; 4 Research Institute for Bioscience Products & Fine Chemicals, Ajinomoto Co., Inc., Kawasaki, Japan

**Keywords:** Translational research, Diagnostic markers, Olfactory receptors, Neural ageing

## Abstract

Olfactory mucus contributes to the specific functions of the olfactory mucosa, but the composition and source of mucus proteins have not been fully elucidated. In this study, we used comprehensive proteome analysis and identified lipocalin 15 (LCN15), a human-specific lipocalin family protein, as an abundant component of the olfactory mucus. Western blot analysis and enzyme-linked immunosorbent assay (ELISA) using a newly generated anti-LCN15 antibody showed that LCN15 was concentrated in olfactory mucus samples, but not in respiratory mucus samples. Immunohistochemical staining using anti-LCN15 antibody revealed that LCN15 localized to the cytokeratin 18-positive Bowman's glands of the olfactory cleft mucosa. Quantitative image analysis revealed that the area of LCN15 immunoreactivity along the olfactory cleft mucosa significantly correlated with the area of neuron-specific Protein-Gene Product 9.5 (PGP9.5) immunoreactivity, suggesting that LCN15 is produced in non-degenerated areas of the olfactory neuroepithelium. ELISA demonstrated that the concentration of LCN15 in the mucus was lower in participants with normal olfaction (≥ 50 years) and also tended to be lower in patients with idiopathic olfactory loss (≥ 50 years) than in participants with normal olfaction (< 50 years). Thus, LCN15 may serve as a biomarker for the activity of the Bowman’s glands.

## Introduction

Olfactory perception starts when odorants entering the nasal cavity bind to olfactory receptors (ORs), expressed by olfactory receptor neurons (ORNs) in the olfactory cleft^1^. Before binding, odorants must dissolve into the olfactory mucus for transport to the ORs. The function of olfactory mucus is thought to include (1) protection of the olfactory cilia^2^, (2) transport and concentration of odorants via odorant binding proteins (OBPs)^3,4^, (3) prevention of mucosal and subsequent brain infection through the secretion of antimicrobial proteins^5,6^, (4) biochemical detoxification through biotransformation enzymes^7^, and (5) metabolism of odorant molecules^8,9^. Olfactory mucus secreted by the Bowman’s glands thus must contain specific molecules to achieve these functions.

Previous studies have analyzed the olfactory mucosa transcriptome and the olfactory mucus proteome in both animals and humans. Lipocalin (LCN) family proteins, which include OBPs, have received close attention due to their ability to bind with small hydrophobic molecules, playing potential roles in chemical communication^10–13^. In mammals, OBPs are abundantly expressed in the olfactory mucosa and are thought to take part in odorant transport, concentration, selection, and elimination^4,14–17^. Briand et al.^18^ detected OBP2 in human olfactory mucus, and Debat et al.^19^ also identified OBP2a and OBP2b during proteome analysis of olfactory mucus. However, neither Wolf et al.^20^ nor Yoshikawa et al.^21^ identified these proteins in the olfactory mucus. Instead, Yoshikawa et al. detected another lipocalin family protein, LCN15^21^. In transcriptome analysis of the human nasal mucosa by Olender et al.^14^, the gene expression of OBPs in the olfactory mucosa was very low, while LCN1 and 15 were more highly expressed in the olfactory mucosa than in the respiratory mucosa. In spite of these recent findings regarding LCN in the human olfactory system, the source and distribution of these proteins in the olfactory mucosa have not been examined in humans, and the effects of aberrant expression on olfactory function have not been fully addressed.

The first aim of this study was to perform a comprehensive analysis of the composition of human olfactory mucus using proteomics and compare it against that of respiratory mucus, using two different sampling methods. Unexpectedly, none of the expected OBPs were detected in olfactory mucus samples. Instead, LCN15 was specifically concentrated in the olfactory mucus. Therefore, we examined the localization of LCN15 in the human olfactory mucosa by immunohistochemistry using a newly raised and purified anti-LCN15 antibody. We also examined the relationship between the mucus LCN15 levels and olfactory function in the study participants. To assess the possible functional roles of LNC15 in the olfactory mucosa, we examined the effects of LCN15 supplementation on OR activation by odorants and on bacterial growth.

## Materials and methods

### Human subjects

For collection of nasal mucus, both individuals with normal olfaction and patients with olfactory impairment were recruited between November 2018 and December 2019. Participants were enrolled in response to an advertisement at the University of Tokyo Hospital and were compensated for their time and effort. Participants included patients, their families, and university hospital employees. Exclusion criteria included severe nasal deformity that would impair insertion of a sponge into the nasal cavity. Once enrolled, the participants completed medical history questionnaires to collect demographic data such as age, gender, and information on medical comorbidities and past/current smoking habits. These characteristics are summarized in Table 1.

**Table 1:** LCN15 concentration in olfactory cleft lavage fluid.

	Normal olfaction, < 50 years (n = 20)	Normal olfaction, ≥ 50 years (n = 14)	Idiopathic olfactory impairment, < 50 years (n = 4)	Idiopathic olfactory impairment, ≥ 50 years (n = 11)	*P*
Age range (years)	26–48	63–84	47–49	60–83	NA
Age (years), mean ± SD	35.0 ± 6.9	76.7 ± 5.8	48.0 ± 1.2	72.3 ± 7.3	NA
Sex (female/male), n	11/9	6/8	2/2	5/6	0.91*
Non-smoker/current or former smoker, n	20/0	9/5	3/1	6/5	0.014*
T&T recognition threshold	≤ 2.0	≤ 2.0	5.8 ± 0.0 (5.8)	4.9 ± 1.0 (2.8–5.8)	NA
LCN15 concentration mean ± SD (ng/2.5 μg protein)	23.6 ± 12.3	14.5 ± 5.3	19.1 ± 7.0	14.8 ± 2.8	0.03**

*NA* not assessed.

*chi-square test.

**one-way analysis of variance (ANOVA).

Diagnosis of olfactory disorders was based on history, endoscopic inspection of the nasal cavity, CT scanning, and olfactory tests. Specific causes of olfactory disorders (i.e., allergy, chronic rhinosinusitis, postviral olfactory disorder, and head trauma) were excluded by these modalities. If patients exhibited olfactory decline, but the cause could not be specified by those modalities, the olfactory dysfunction was diagnosed as idiopathic.

### Human nasal tissue

To examine the localization of LCN15 in the human nasal mucosa, archival nasal tissues containing the olfactory cleft and cribriform plate (n = 10) were used. These surgical specimens were collected from patients who underwent resection of the skull base, including the cribriform plate, for the treatment of tumors, such as olfactory neuroblastoma. Patient information is shown in Supplementary Table 1. According to the Institutional Review Board instructions, we obtained written informed consent for the study. In the cases of dead patients or in cases where contact information could not be obtained at the time of study entry, we offered the opportunity to opt-out to the patients or their descendants on our website. The initial number of patients enrolled was 23, and 13 patients were excluded from the study, because their archival mucosa specimens were not suitable for analysis, either due to mechanical artifact during surgical removal of the tissue, or due to massive tumor extension into the olfactory cleft. Unfortunately, olfactory test data were not available for any patient because the analysis was retrospective and the patients had been treated for malignant tumors, not olfactory dysfunction. We therefore included all specimens in which the nasal septum, ethmoid tegmen, and middle turbinate were anatomically identified and the condition of the mucosa was acceptable for histological evaluation.

All specimens were decalcified and embedded in paraffin. Sections, 4 μm in thickness, were cut and mounted on MAS-coated slides (Matsunami Glass, Osaka, Japan).

### Olfactory testing

Olfactory acuity was defined as the average of the recognition thresholds of T&T olfactometry^22^, with a slight modification. T&T olfactometry is the standard smell test array in Japan and uses the following five odorants: A: β-phenyl ethyl alcohol, which smells like rose; B: methyl cyclopentenolone, which smells like burnt sugar; C: isovaleric acid, which smells like sweat; D: γ-undecalactone, which smells like peach; and E: skatole, which smells like garbage (Daiichi Yakuhin Sangyo, Tokyo, Japan). The concentrations of each odorant ranged over eight degrees of intensity (− 2 to 5), except for cyclopentenolone, which ranged over seven degrees (− 2 to 4). During the test, patients sniffed strips of paper dipped in various concentrations of the five odorants. The recognition threshold was defined as the lowest concentration at which the odor could be identified. The recognition thresholds of the five odorants were averaged.

We began the presentation of each odor at level 2, and when the participants could identify all the five odorants at level 2, they were categorized as having normal olfaction, according to previous studies^23^. If they could not identify any of the odorants, the concentration of odorants was increased until they could identify the odorants. If they could not identify at the highest concentrations (5 in A, C, D, and E, and 4 in B), their threshold was calculated as 6 for A, C, D, and E, and 5 for B. The mean ± SD of the average recognition threshold of A–E in each group is shown in Table 1.

### Collection and preparation of human nasal mucus

1. Irrigation of the superior nasal cavity with saline

To obtain mucus from the superior part of the nasal cavity, including the olfactory cleft, we irrigated the olfactory cleft with saline^8^. The participants were placed in the prone position, with their knees bent and their forehead on the floor (Supplementary Fig. 1). One milliliter of saline at 37 °C was introduced into the nostril on one side and left for 1 min. The participant then stood up, and the liquid that exited the nostril was collected into a tube, immediately frozen in liquid nitrogen, and stored at − 80 °C for storage. These samples are referred to as olfactory cleft lavage fluid (OCLF) in the following sections.

(2) Insertion of the surgical sponge into the olfactory cleft

Mucus was collected with sponges according to previous studies^21,24^ with some modifications. Following the application of topical lidocaine, the sponge portion of an eye spear (Ivalon® Lasik Eye sponge, New London, CT, USA) was placed in the left olfactory cleft between the middle turbinate and superior nasal septum under endoscopic or microscopic inspection and held there for 10 min. The sponge was then removed from the nose and placed in a 1.5 mL tube with a cassette filter for collection (Centrifugal Filter Units UFC30SV00, Merck Millipore, County Cork, Ireland). The tube was centrifuged at 9550 × *g* for 5 min to separate the mucus. The collected mucus was frozen in liquid nitrogen at − 80 °C until use. These samples are referred to as olfactory cleft mucus collected by surgical sponge (OCMS) in the following sections.

Each participant underwent both the olfactory mucus sampling procedures. One side of the nasal cavity was used for irrigation, and the other side was used for sponge insertion. The decision on which side was used for irrigation/sponge insertion was made based on the visibility of the olfactory cleft.

(3) Respiratory mucus

Surgical sponges (Merocel®, Medtronic Japan, Tokyo, Japan), 1 mm in diameter and 2 cm long, were placed for 10 min in both the right and left nostrils to absorb mucus from the respiratory mucosa. The sponges were then removed from the nose and placed in a tube for collection, as in the OCMS procedure. The tube was centrifuged at 9550 × *g* for 5 min to separate the mucus. The collected mucus was frozen in liquid nitrogen and stored at − 80 °C until use.

### Proteome analysis

We aimed to detect the following human LCN family proteins in the mucus samples: LCN1, LCN2, LCN6, LCN8, LCN9, LCN10, LCN12, LCN15, OBP2A, OBP2B, alpha-1-microglobulin/bikunin precursor, apolipoprotein D, apolipoprotein M, complement component C8 gamma chain, alpha-1-acid glycoprotein 1, alpha-1-acid glycoprotein 2, progestagen-associated endometrial protein, prostaglandin D synthetase, and retinol-binding protein. The mucus samples (OCLF and respiratory mucus, n = 3; from participants with normal olfaction < 50 years, age range 35–48 years, mean age 43.3 years) digested with trypsin were analyzed using nano liquid chromatography tandem mass spectrometry (LC/MS/MS) analysis (Supplementary Materials and Methods). The resulting digested peptide mixtures were analyzed using an Orbitrap Fusion wETD (Thermo Fisher Scientific, San Jose, CA, USA) equipped with an Easy-nLC 1000 (Thermo Fisher Scientific). The resultant LC/MS/MS data were searched against the Swiss-Prot Human Database (2017.7) using the Mascot (version 2.6.0, Matrix Science Inc., London, UK) search engine. Scaffold (version 4.8.3, Proteome Software, Inc., Portland, OR, USA) was used to integrate the proteomics data and compare the total spectrum counts. The thresholds for protein false discovery rate (FDR), peptide FDR, and number of peptides per protein were set to 1.0%, 0.1%, and 2%, respectively.

### Preparation of anti-LCN15 antibody

Anti-LCN15 polyclonal antibody was developed by immunizing rabbits with the recombinant LCN15 protein at Eurofin Genomics Co. Inc. (Tokyo, Japan). Whole serum was extracted from the rabbits and further purified using an affinity column of LCN15.

### Western blot analysis

A mucus sample containing 1.82 μg protein was mixed with 4 × Lithium Dodecyl Sulfate sample buffer (Life Technologies) and fractionated through sodium dodecyl sulfate–polyacrylamide gel electrophoresis (SDS-PAGE) using 10 or 12% polyacrylamide NuPAGE Bis–Tris gels (Thermo Fisher Scientific, Tokyo, Japan) according to the manufacturer’s protocol. The proteins were transferred to polyvinylidene fluoride (PVDF) membranes using an iBlot system (Life Technologies). The membranes were subjected to immunoblotting using the iBind system (Life Technologies). The primary antibody was anti-LCN15 polyclonal (1:1,000), and the secondary antibody was horseradish peroxidase (HRP)-conjugated anti-rabbit IgG (1:13,000, KPL, MA, USA). The blots were developed with SuperSignal West Dura Extended Duration Substrate (Thermo Scientific) and captured using an Amersham Imager 600 (GE Healthcare).

### Enzyme-linked immunosorbent assay (ELISA)

The LCN15 sandwich ELISA kit was prepared by MABEL Inc. (Kyoto, Japan). The microplates were coated with the capture antibody, a custom-made anti-LCN15 polyclonal antibody at 1 μg/mL in phosphate-buffered saline (PBS), and incubated overnight at 4 °C, rinsed with wash buffer (0.05% Tween-20 in PBS), and blocked with 1% bovine serum albumin in PBS at 37 °C for 30 min. Standard dilutions of LCN15 were made in a range from 3.125 to 100 ng/mL, and mucus samples were adjusted to 2.5 μg/mL protein by dilution in PBS. After washing with wash buffer, biotinylated anti-LCN15 (1 μg/mL) was added to each well and incubated at 37 °C for 30 min. The plate was washed, and HRP-streptavidin (1:10,000) was added and incubated at 37 °C for 30 min. The plate was again washed, and tetramethylbenzidine substrate was added to each well and incubated at 23 °C for 15 min in the dark. Finally, to stop color development, 1 M H_2_SO_4_ was added, and absorbance at 450 nm was measured using a microplate reader (Bio-Rad, CA, USA).

The purified LCN15 standard was produced using a gram-positive *Corynebacterium glutamicum* protein expression service (CORYNEX®) provided by Ajinomoto Co., Inc. (Tokyo, Japan).

### Immunohistochemistry

To examine the localization of LCN15 in the olfactory mucosa and identify its association with neuroepithelium distribution, we used immunohistochemistry for LCN15, Protein-Gene Product 9.5 (PGP9.5), and cytokeratin 18 (CK18). PGP9.5 is a pan-neuronal marker used to visualize ORNs in human olfactory mucosa^25^. CK18 is an epithelial marker expressed in supporting cells, Bowman’s glands^25^, and epithelial cells of the respiratory mucosa^26,27^.

For single immunostaining with the anti‐LCN15, PGP9.5, and CK18 antibodies, sections were deparaffinized and then rehydrated through a xylene and ethanol series. The sections were then immersed in Antigen Retrieval Solution (DAKO S1700; Agilent Technology, Tokyo, Japan) and autoclaved at 121 °C for 20 min to retrieve antigens.

After antigen retrieval, endogenous peroxidase activity was blocked by treatment with 3% hydrogen peroxide in methanol for 10 min at 23 °C. The sections were then incubated for 10 min with a blocking solution (Blocking-One Histo, Nacalai Tesque Inc., Tokyo, Japan) at 23 °C in order to reduce nonspecific antibody binding, followed by incubation with one of the following rabbit antibodies at 23 °C for 1 h: anti-PGP9.5 antibody (DAKO; Agilent Technology, Tokyo, Japan; 1:500 in antibody dilution solution [Tris-buffered saline with 0.1% of Tween 20 and 5% blocking solution]), anti-CK18 antibody (Abcam, Tokyo, Japan; 1:500 in antibody dilution solution), and anti-LCN15 antibody (1:3,000 in antigen dilution solution). After several washes in PBS (pH 7.4), the sections were incubated for 30 min at 23 °C with HRP-conjugated anti-rabbit IgG secondary antibody (Simple Stain MAX PO [R], ready-to-use; Nichirei, Tokyo, Japan). After more washes with PBS (pH 7.4), immunoreactivity was examined using diaminobenzidine (DAB) staining (Simple Stain DAB, ready-to-use; Nichirei) according to manufacturer instructions. After washing with distilled water, the sections were counterstained with hematoxylin, dehydrated, and mounted. As a negative control, the primary antibody was omitted from the reaction. There was no obvious labeling corresponding to immunostaining with the primary antibody.

For double immunostaining with the anti-LCN15 and anti-PGP9.5 antibodies, the rehydrated sections were immersed in Antigen Retrieval Solution, autoclaved at 121 °C for 20 min, processed to block endogenous peroxidase activity and nonspecific antibody binding as described above, and then incubated with rabbit anti-LCN15 antibody at 4 °C overnight. After washing with PBS (pH 7.4), the sections were incubated with HRP-conjugated anti-rabbit IgG secondary antibody for 30 min at 23 °C. LCN15 immunoreactivity was visualized with DAB staining. After further washing with distilled water, the sections were placed in a citrate buffer solution (pH 6.0, DAKO S2031; Agilent Technology, Tokyo, Japan) and autoclaved at 121 °C for 5 min to deplete the antigenicity of the anti-LCN15 primary antibody to the secondary antibody and to inactivate the enzymatic activity of HRP binding to the anti-rabbit IgG antibody. After several washes in PBS (pH 7.4), the sections were incubated for 1 h at 23 °C with rabbit anti-PGP9.5 antibody. After several washes with PBS (pH 7.4), the sections were incubated for 30 min at 23 °C with HRP-conjugated anti-rabbit IgG antibody. After additional washes with PBS (pH 7.4), immunoreactivity was measured using a Vector SG kit (Vector Laboratories, Burlingame, CA, USA) according to the manufacturer’s instructions. After washing with distilled water, the sections were dehydrated and mounted. Preliminary experiments were conducted to confirm that autoclaving of the sections after DAB staining depleted both further secondary anti-rabbit IgG antibody binding to anti-LCN15 antibody and the enzymatic activity of HRP before autoclaving.

Microscopic images (Figs. 2 and 3 and Supplementary Figs. 3, 4) were acquired using a BZ-X700 microscope (Keyence, Osaka, Japan).

### Quantitative image analysis

The area distribution of PGP9.5 and LCN15-immunoreactivity in the olfactory cleft mucosa was analyzed quantitatively using serial sections stained with anti-CK18 antibody and double-stained with anti-LCN15 and anti-PGP9.5 antibodies at GeneticLab (Sapporo, Japan).

First, the whole area of CK18 stained and LCN15/PGP9.5 double-stained sections from the same participant were scanned using a Brightfield slide scanner (Leica Microsystems K.K., Scanscope AT Turbo) at 20 × magnification. The images were saved as SVS files.

The location of the olfactory cleft mucosa was identified using CK18-stained sections, and rectangular areas (1 mm × 1 mm) for analysis were set serially along the mucosal lining of the olfactory cleft using the rectangle tool in HALO V2.3.2089.70 (Indica Lab). The same sets of rectangular areas were placed on serial sections double-stained for LCN15 and PGP9.5. The number of set areas, that is, the mucosal length of the olfactory cleft for each specimen was variable depending on the surgical procedures performed on the patients, but 2–49 serial areas for analysis were set in each section. The areas of PGP9.5 and LCN15 immunoreactivity were measured using the HALO Area Quantification V1.0 algorithm. In the area setting, the mucosal area showing phenomena such as epithelial peeling and nonspecific staining was excluded from analysis by the exclusion tool of HALO.

To examine the spatial dynamics of PGP9.5- and LCN15-immunoreactive areas, we divided the olfactory cleft mucosa of each sample into four zones (dorsomedial, dorsolateral, ventromedial, and ventrolateral) and analyzed each separately. We then compared the areas of PGP9.5- and LCN15-immunoreactivity in each zone.

To examine the spatial distribution of LCN15-negative, CK18-positive submucosal glands, the LCN15- and CK18-positive glands were manually counted in each zone described above. Because all of the LCN15-positive glands were also CK18-positive, the number of LCN15-positive glands was subtracted from the number of CK18-positive glands to count LCN15-negative, CK18 positive glands. We compared the ratio of the number of LCN15-negative glands to the total number of CK18-positive glands with the area of PGP9.5 immunoreactivity in each of the four zones.

### Statistical analysis

As shown in Table 1, sex distribution and smoking status among the participants was evaluated using the chi-square test. The concentration of LCN15 in the OCLF was statistically analyzed by one-way analysis of variance (ANOVA), followed by Tukey’s post-hoc test with correction for multiple tests, using the R software (version 3.6.3) to compare each group. In Table 2, the value in each cell indicates the total mass spectrum counts used to identify proteins. *P* values were evaluated using a non-paired *t*-test. In Fig. 5, the correlation between the area of PGP9.5 immunoreactivity and that of LCN15 immunoreactivity in each rectangular field, as well as for each patient, was statistically examined using Spearman’s rank correlation. The zonal differences in correlation between PGP9.5- and LCN15-immunoreactive areas (Supplementary Fig. 5) and the correlation between the ratio of the number of LCN15-negative/CK18-positive glands and the PGP9.5-immunoreactive area in the olfactory cleft mucosa (Supplementary Fig. 6) were statistically examined using the Spearman’s rank correlation. Differences were considered statistically significant at *P* < 0.05.

**Table 2:** Lipocalin counts in proteome analysis of mucus.

Mucus type	Olfactory			Respiratory			P*
Subject no	#1	#2	#3	#1	#2	#3	
NGAL (lipocalin-2)	22	23	22	12	10	13	0.0003
Lipocalin-15	348	172	497	11	9	16	0.025
Lipocalin-1	58	1	3	92	26	6	0.554
Alpha-1-microglobulin/bikunin precursor	0	0	0	3	1	0	NA^†^
Apolipoprotein D	0	2	0	4	1	1	NA^†^
Retinol-binding protein	0	3	0	6	6	1	NA^†^

The value in each cell indicates the total mass spectrum counts used to identify proteins.

*non-paired *t* test.

^†^NA: not assessed because of low abundance.

### Image presentation

Digital images of the photographs (Figs. 1, 2, 3 and 4 and Supplementary Figs. 1–4) were processed with Adobe Photoshop 2022 (Adobe, Tokyo, Japan), adjusting only for brightness, contrast, and color balance. Graph charts (Fig. 5 and Supplementary Figs. 5–9) were made with GraphPad Prism 5 (GraphPad Software, La Jolla, CA).

**Figure 1:**
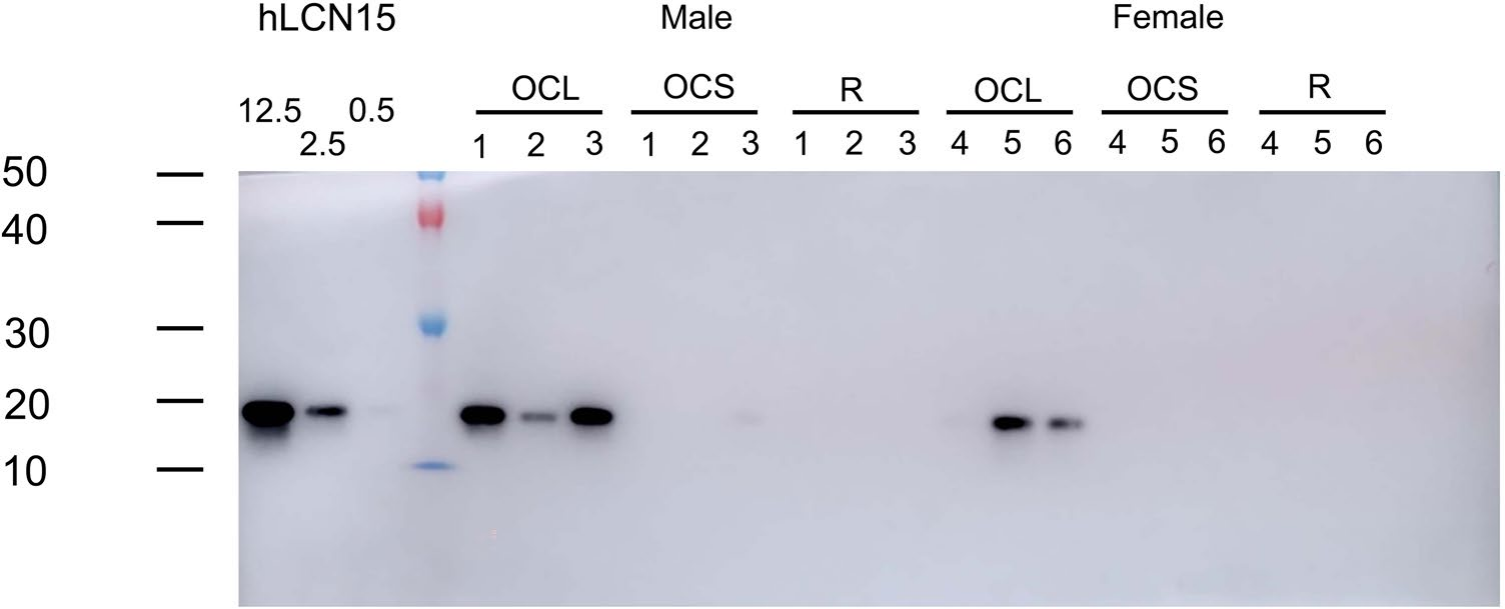
Western blot analysis of LCN15 in the olfactory and respiratory mucus using rabbit anti-LCN15 antibody. Respective immunoblots of samples of olfactory cleft lavage fluid (OCLF), olfactory cleft mucus collected by surgical sponge (OCMS) and respiratory mucus (R) from six participants with normal olfaction (3 males [No. 1–3] and 3 females [No. 4–6]). A mucus sample containing 1.82 μg protein was loaded in each lane. The three lanes in the left end show recombinant LCN15 protein (12.5, 2.5 and 0.5 ng/mL) as a positive control.

**Figure 2:**
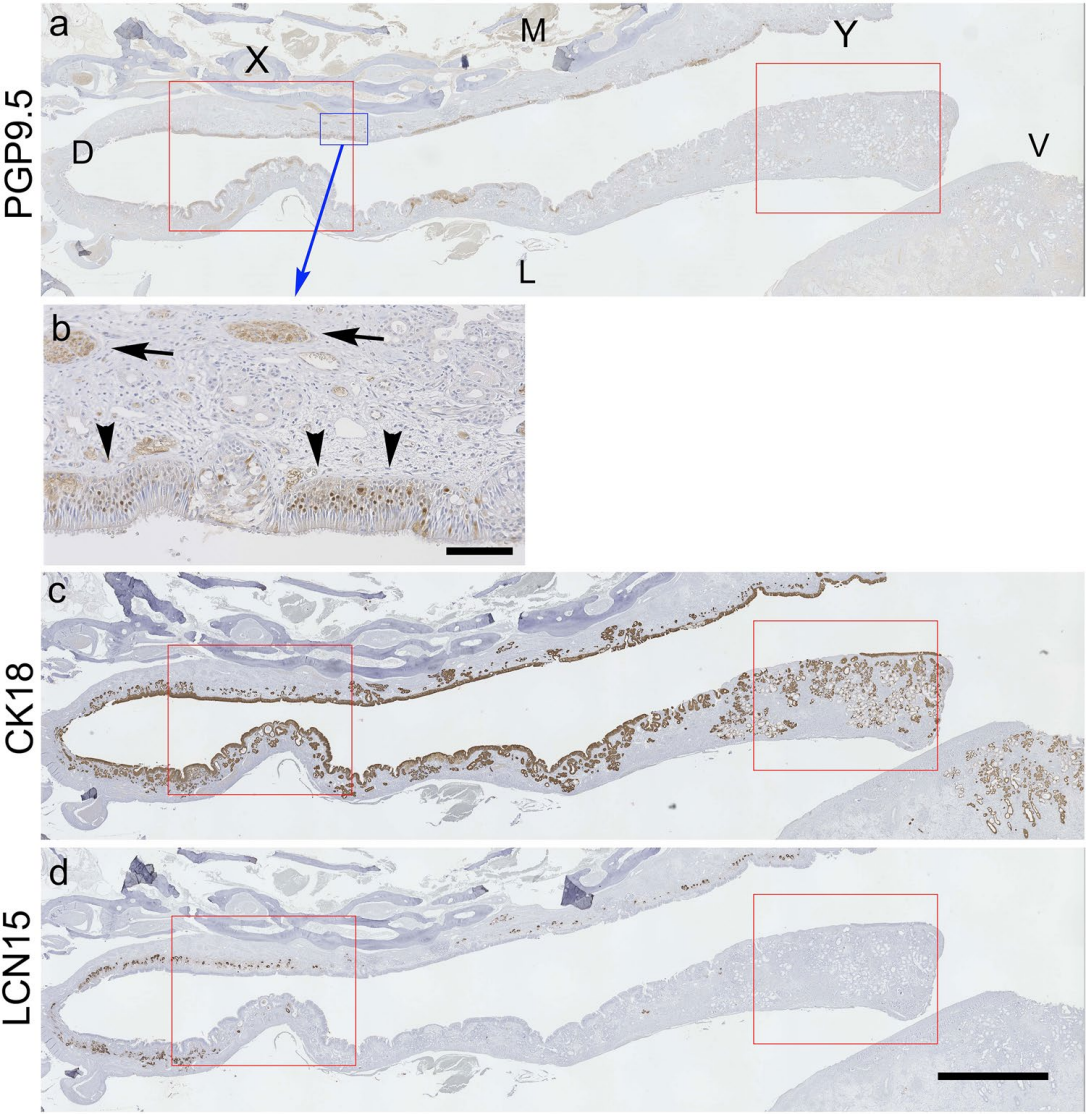
Immunohistochemical staining of human olfactory cleft mucosa sections with rabbit anti-PGP9.5 antibody (**a** and **b**), rabbit anti-cytokeratin 18 antibody (**c**), and rabbit anti-LCN15 antibody (**d**). A, C, D: D, V, M, and L in A indicate dorsal, ventral, medial, and lateral zones of the nasal cavity, respectively. Left and right sides of the image correspond to superior and inferior ends of the olfactory cleft. Red rectangles (X and Y) indicate microscopic fields, shown as a magnified view in Supplementary Fig. 3. (**a**): PGP9.5 immunoreactivity is observed in sparse island areas of the olfactory mucosa area. (**b**): Magnified view of the field indicated by the blue rectangle in **a**. PGP9.5-positive ORNs are observed in the epithelium (black arrowheads). In the lamina propria, PGP9.5-positive olfactory nerve bundles are shown by the black arrows. (**c**): CK18 immunoreactivity in the epithelium and submucosal glands of the olfactory cleft mucosa. (**d**): LCN15 immunoreactivity in the submucosal glands, mainly in the superior end of the cleft. Scale bar in **b** = 100 μm and in **d** (also applies for **a** and **c**) = 2 mm.

**Figure 3:**
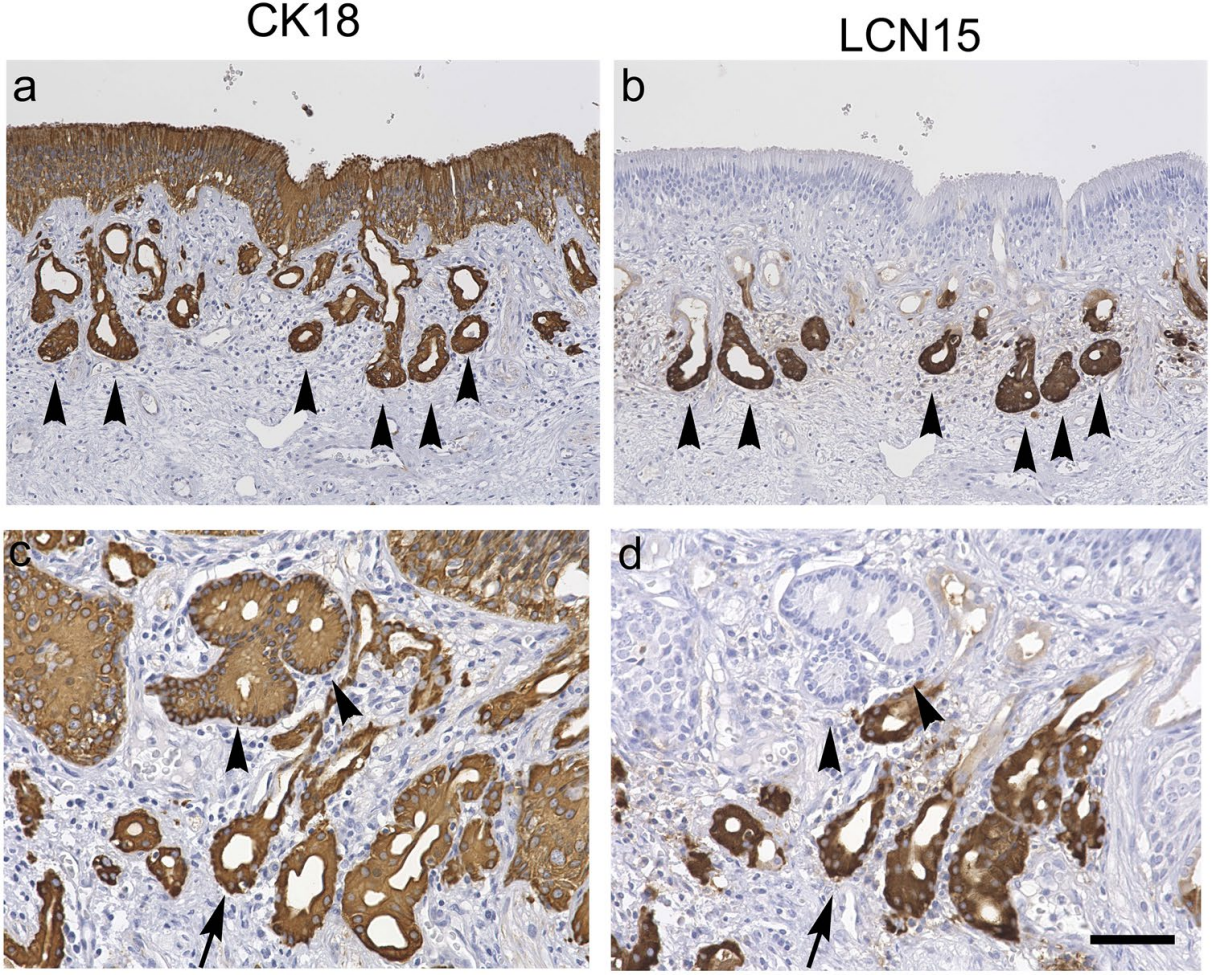
(**a**,**b**): Magnified views of the serial human olfactory mucosa sections, stained with rabbit anti-cytokeratin 18 antibody (**a**) and rabbit anti-LCN15 antibody (**b**). Black arrows in **a** and **b** indicate identical acini of the glands. CK18 immunoreactivity was observed in the whole thickness of the epithelium, ducts of the submucosal glands, and acini of the glands (**a**). In comparison with A, LCN15 immunoreactivity is observed only in the acini of the submucosal glands (**b**). (**cd**): LCN15-negative glands in the superior area of the olfactory cleft. Magnified views of the serial human olfactory mucosa sections stained with rabbit anti-cytokeratin 18 antibody (**c**) and rabbit anti-LCN15 antibody (**d**). A black arrow and arrowheads in **c** and **d** indicate identical acini of the glands. LCN15-negative glands appeared to have less dense CK18 staining, and the nuclei of the acinar cells were located adjacent to the basal membrane. Scale bar in **d** (also applies to **a**–**c**) = 100 μm.

**Figure 4:**
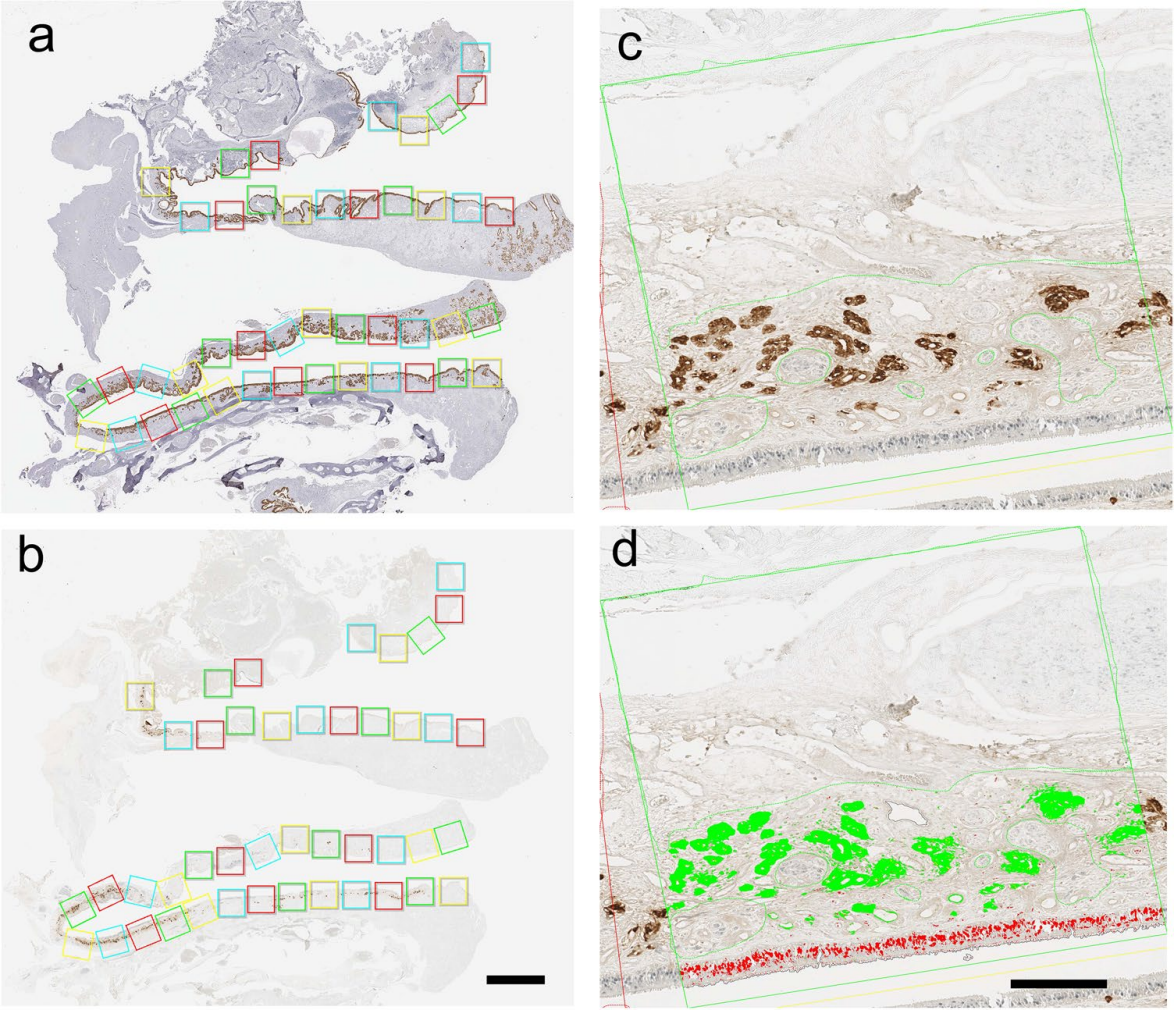
Quantitative image analysis of the area for PGP9.5 and LCN15 immunoreactivity in the olfactory cleft mucosa. (**a**): Olfactory cleft mucosal lining with intact epithelium was morphologically identified using sections stained for cytokeratin 18. Using these sections, rectangle areas (1 × 1 mm) for analysis were serially set along the mucosal lining. (**b**): The same rectangular areas used in **a** were set on the serial sections stained for PGP9.5 and LCN15 for analysis. (**c**): A magnified view of the image in **b**. The immunoreactivity for PGP9.5 and LCN15 is visualized in gray and brown colors, respectively. (**d**): Areas of PGP9.5 and LCN15-immunoreactivity in each analysis area were automatically selected and marked in red (PGP9.5) and green (LCN15), respectively, by setting the color threshold. Scale bars in B and D are = 2 mm and 200 μm, respectively.

**Figure 5:**
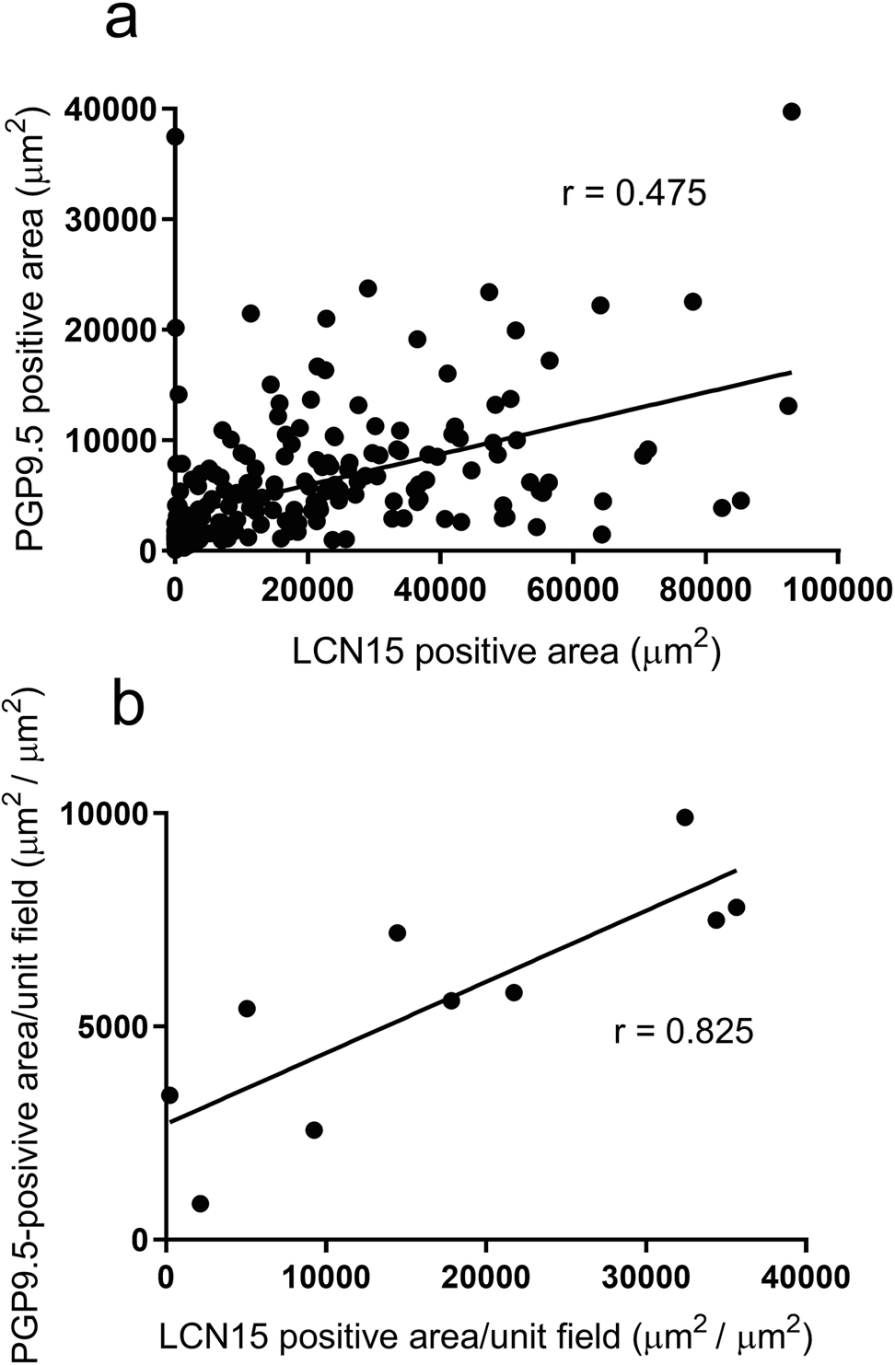
Distribution correlation of LCN15-immunoreactive and PGP9.5-immunoreactive areas in the olfactory cleft mucosa. (**a**): The total area of LCN15 immunoreactivity and that of PGP9.5 immunoreactivity in each rectangle field were significantly correlated (Spearman’s rank correlation, r = 0.475; *P* < 0.0001). (**b**): The total area of LCN15 immunoreactivity along the unit field of olfactory cleft mucosal lining in each participant was significantly correlated with that of PGP9.5 immunoreactivity (Spearman’s rank correlation, r = 0.825; *P* = 0.0033).

### Ethics approval and consent to participate

This study was approved by the study review committee at Ajinomoto Co., Inc. (no. 2018–009, 2018–026), the Institutional Review Board at the University of Tokyo Hospital (no. 2018092NI, 2019073NI), and Mie University Hospital (no. H2019-161). All procedures were conducted in accordance with the Declaration of Helsinki. All participants who provided nasal mucus samples provided written informed consent prior to participation in this study.

## Results

### Proteome analysis of olfactory and respiratory mucus

OCLF and respiratory mucus from participants with normal olfaction (aged < 50 years; n = 3) were subjected to proteome analysis. The top 10 most abundant proteins in the OCLF and respiratory mucus are listed in Supplementary Table 2; polymeric immunoglobulin receptor, immunoglobulin heavy constant alpha 1, albumin, lactotransferrin, lysozyme C, BPI fold-containing family B member 1, and prolactin-inducible protein are listed in both mucus proteomes, while LCN15, fibronectin, and transcobalamin-1 are listed only in the list of OCLF proteome.

We were interested in identifying OBPs, which are thought to bind odorants and transport them to the ORs^4^. Of the family proteins, we identified LCN1, LCN2, LCN15, alpha-1-microglobulin/bikunin precursor, apolipoprotein D, and retinol-binding protein (Table 2). OBPs were not detected in OCLF. LCN15 was the second most abundant protein identified in OCLF (Supplementary Table 2) and its levels were significantly higher in OCLF than in respiratory mucus (Table 2; *P* = 0.025). LCN15 was therefore examined in the following analysis.

### Production and specificity of polyclonal anti-LCN15 antibody

We prepared recombinant LCN15 protein using the hLCN15 expression vector in *Escherichia coli*. The protein was purified from cell lysates. SDS-PAGE of the recombinant protein showed a single band at approximately 18 kDa, corresponding to LCN15 (Supplementary Fig. 2a), and a mass of 18,319 Da was observed by intact protein mass spectrometry analysis (Supplementary Fig. 2b). The purity of the recombinant LCN15 was further confirmed by LC/MS/MS peptide mapping analysis, where the sequence coverage of LCN15 was 95%.

Next, we prepared rabbit polyclonal anti-LCN15 antibody using recombinant LCN15 protein as an immunogen. To examine the specificity of the anti-LCN15 antibody, western blotting was performed (Fig. 1). Our anti-LCN15 antibody recognized the band at the predicted molecular weight of LCN15 (18 kDa) in the OCLF and OCMS, suggesting the specificity of the antibody.

### Comparison of LCN 15 levels between OCLF, OCMS, and respiratory mucus

On western blot analysis, anti-LCN15 antibody recognized clear bands in five of six OCLF samples and a faint band in one OCMS sample but did not recognize any in the respiratory mucus samples (Fig. 1). This finding confirmed our proteome analysis, suggesting that LCN15 is richly expressed in olfactory mucus but not in respiratory mucus. We used ELISA to quantitatively compare the levels of LCN15 in the OCLF, OCMS, and respiratory mucus samples from eight participants with normal olfaction (age range 33–43 years, mean 37.3 ± 3.8 years). LCN15 concentration was measured (μg) per milliliter of each mucus sample, as well as (ng) per 2.5 μg total protein in the mucus samples.

This analysis confirmed that olfactory mucus contained a higher concentration of LCN15 than the respiratory mucus (Table 3). The total protein concentration in the OCLF, OCMS, and respiratory mucus samples was 562 ± 178 μg/mL, 8980 ± 3500 μg/mL, and 7835 ± 4490 μg/mL, respectively. LCN15 concentration in the OCLF, OCMS, and respiratory mucus samples was 2.94 ± 1.29 μg/mL, 5.07 ± 3.79 μg/mL, and 0.94 ± 1.00 μg/mL, respectively. The OCLF and OCMS samples showed approximately threefold and fivefold higher concentrations of LCN15 than the respiratory mucus samples.

**Table 3:** Total protein and LCN15 concentration in the mucus samples.

	Olfactory cleft lavage fluid (OCLF) (n = 8)	Olfactory cleft mucus collected by sponges (OCMS) (n = 8)	Respiratory mucus (n = 8)
Protein concentration (μg/mL, mean ± SD)	562 ± 178	8980 ± 3500	7835 ± 4490
LCN15 concentration (μg/mL, mean ± SD)	2.94 ± 1.29	5.07 ± 3.79	0.94 ± 1.00
LCN15 content (ng/2.5 μg protein in mucus, mean ± SD)	14.29 ± 8.12	1.37 ± 0.97	0.24 ± 0.21

Unexpectedly, LCN15 was selectively concentrated in the OCLF samples (Table 3). LCN15 levels relative to total protein in the OCLF samples were approximately tenfold and 60-fold higher than those in the OCMS and respiratory mucus samples (14.29 ± 8.12 vs. 1.37 ± 0.97 vs. 0.24 ± 0.21 ng/2.5 μg protein), respectively. Although the total protein concentration in OCLF was 0.06-fold higher than that in OCMS and 0.07-fold higher than that in respiratory mucus (562 ± 178 vs. 8980 ± 3500 vs. 7835 ± 4490 μg/mL), LCN15 concentration in OCLF was 0.6-fold and 3.1-fold greater than those in OCMS and respiratory mucus (2.94 ± 1.29 vs. 5.07 ± 3.89 vs. 0.94 ± 1.00 μg/mL).

### Immunohistochemical localization of LCN15 in the human nasal mucosa

To examine the localization of LCN15 in the nasal mucosa, we stained the human nasal mucosa containing olfactory cleft with anti-LCN15 antibody and compared the localization of its immunoreactivity with those of PGP9.5 (neuronal marker) and CK18 (epithelial marker) (Fig. 2, sample from patient #5). PGP9.5-, CK18-, and LCN15-immunoreactivities were similarly observed in all 10 human tissue samples, despite variations in the tissue integrity due to surgically induced mechanical artifacts. By immunostaining with an anti-PGP9.5 antibody, we characterized the region of the olfactory neuroepithelium as containing sparse, island-like areas (Fig. 2a, b, Supplementary Fig. 3a, d). Immunostaining with an anti-CK18 antibody was used to visualize the neuroepithelium, respiratory epithelium, and submucosal glands beneath the olfactory and respiratory epithelia (Figs. 2c and 3a, c, Supplementary Fig. 3b, e). In contrast, immunostaining with anti-LCN15 polyclonal antibodies revealed the localization of LCN15 in the proximal region of the Bowman's glands, which were also CK18-positive and located mainly in the middle region of the lamina propria in the olfactory cleft mucosa (Figs. 2c, d and 3a–d, Supplementary Fig. 3b, c, e, f). The superior region of the lamina propria, which was adjacent to the basal membrane, was LCN15-negative and slightly less CK18-positive, even in the Bowman's glands, compared with the inferior region (Fig. 3a–d, Supplementary Fig. 3b, c, e, f). These results suggested that the acinar cells of the Bowman's glands in the middle region of the lamina propria of the olfactory cleft mucosa were the source of the secreted LCN15.

Although the distributions of LCN15 and PGP9.5 immunoreactivity were not completely identical in the mucosal area, LCN15 immunoreactivity tended to be localized in close proximity to PGP9.5-positive ORNs (Fig. 2a, b, d). The possible association between the distribution of ORNs and that of LCN15-immunoreactive cells is clinically important because it could imply the potential of LCN15 in the olfactory mucus as a non-invasive biomarker to estimate ORN amount in patients with olfactory dysfunction. To address this issue, double-stained sections of olfactory cleft mucosa with anti-LCN15 and anti-PGP9.5 antibodies (Supplementary Fig. 4) were scanned as serial rectangular fields, and the doubly immunopositive areas in each field were automatically marked and calculated (Fig. 4). This analysis revealed a significant correlation between the areas of LCN15 and PGP9.5 immunoreactivity in each field (Fig. 5a; Spearman’s rank correlation, r = 0.475; *P* < 0.0001). Furthermore, the total areas of LCN15 and PGP9.5 immunoreactivity along the unit length of the olfactory cleft mucosal lining in each participant were also significantly correlated (Fig. 5b; Spearman’s rank correlation, r = 0.825; *P* = 0.0033). To further examine the spatial dynamics of PGP9.5 and LCN15 immunoreactivity, we compared the areas of PGP9.5-immunoreactivity and LCN15-immunoreactivity in each zone of the mucosa (see *Quantitative image analysis*; Supplementary Fig. 5). Coefficient of determinations between the two immunoreactive areas were higher in the ventromedial (R^2^ = 0.3985) and ventrolateral (R^2^ = 0.2922) areas than in the dorsomedial (R^2^ = 0.2314) and ventrolateral (R^2^ = 0.1556) areas.

A proportion of CK18-positive glands in the olfactory cleft mucosa was found to be LCN15-negative. To examine the distribution of these glands in detail, the number of LCN15-negative glands was counted and the ratio of LCN15-negative glands/total CK18-positive glands was compared with the area of PGP9.5-immunoreactivity in each of the four zones. Coefficient of determination between the parameters was less than 0.2 in each of the four areas, indicating there was no statistical correlation (Supplementary Fig. 6).

### Comparison of LCN15 concentration between normal olfaction and idiopathic olfactory impairment groups

We compared the LCN15 concentrations in the OCLF samples of patients with normal olfaction and with idiopathic olfactory impairment using ELISA. Each group was further divided into two subgroups based on the age, < 50 years and ≥ 50 years. The LCN15 concentration was lower in the participants with normal olfaction (≥ 50 years) (14.5 ± 5.3 ng/2.5 μg total protein; *P* = 0.048) and also tended to be lower in the patients with olfactory impairment (≥ 50 years) (14.8 ± 2.8 ng/2.5 μg total protein; *P* = 0.090) than in the participants with normal olfaction (< 50 years) (23.6 ± 12.3 ng/2.5 μg total protein) (Table 3, Supplementary Fig. 7). No significant difference was observed in LCN15 concentration between participants with normal olfaction (≥ 50 years) and patients with idiopathic olfactory impairment (≥ 50 years) (*P* = 1.00).

### Effects of LCN15 supplement on OR activity assessed via in vitro luciferase assay

To examine the effects of LCN15 supplementation in culture media on in vitro OR activity by odorant binding, the following six pairs of ORs and their agonists were selected and used in the luciferase assay: OR10G-eugenol, OR5AN1-muscon, OR51E1-isovaleric acid, OR2W1-benzylacetate, OR10G4-ethylvanillin, and OR1A1-carvone. Because no distribution map of human ORs in the entire olfactory mucosa has been established, we chose a set of ORs based on the molecular variability of their ligands. The dose-dependent analysis was performed with 3–100 μM of each odorant supplemented with LCN15 (25 μM) or bovine serum albumin (25 μM) or PBS. In two OR-odorant combinations (ORAN1-muscone and OR51E1-isovaleic acid), half of the maximal effective concentration (EC50) of LCN15 and albumin groups were out of the logEC50 95% confidence index (CI) of the PBS group, suggesting that LCN15 and albumin may affect the odorant perception in these combinations (Supplementary Table 3 and Supplementary Fig. 8). On the contrary, all EC50 values in LCN15 group were within the logEC50 95% CI of the corresponding albumin group, suggesting no statistically significant difference between LCN15 and albumin groups in any OR-odorant combination.

### Effects of LCN15 on bacterial growth

We performed a preliminary functional study to test the bacteriostatic effects of LCN15. We hypothesized that LCN15 may contribute to the prevention of infection in the olfactory mucosa because (1) LCN2 is known to exhibit bacteriostatic effects by binding bacterial siderophores and depriving iron of the bacteria^28^, and (2) olfactory mucosa is a direct infectious pathway from the nasal cavity to the brain, and may therefore have a unique preventive system against bacterial infection. To test the bacteriostatic effect of LCN15, we examined the effect of LCN15 supplementation on the growth of *E. coli* in M9 minimal medium under iron-limiting conditions. After the addition of LCN15 at a concentration of 5 μM, growth inhibition of approximately 35% was observed (Supplementary Fig. 7).

## Discussion

In this study, we used comprehensive proteome analysis and identified LCN15 as an abundant component of the olfactory mucus. Western blot analysis and ELISA showed that LCN15 was more concentrated in the olfactory mucus (OCLF and OCMS) than in the respiratory mucus. ELISA of the OCLF samples also revealed that LCN15 levels were lower in the participants with normal olfaction (≥ 50 years) and also tended to be lower in the patients with olfactory impairment (≥ 50 years) than in the participants with normal olfaction (< 50 years). Immunostaining with anti-LCN15 and anti-CK18 antibodies identified the acinar cells of the Bowman's glands in the middle region of the lamina propria as the source of secreted LCN15. Quantitative image analysis showed that the area of LCN15 immunoreactivity along the olfactory cleft mucosa significantly correlated with the area of neuron-specific PGP9.5 immunoreactivity, suggesting that LCN15 is produced in non-degenerated areas of the olfactory neuroepithelium. These findings indicate that LCN15 may serve as a biomarker for Bowman’s gland activity. This protein may also be clinically useful for non-invasive estimation of human olfactory neuroepithelium amount.

Our proteome analysis confirmed the previously reported findings that the olfactory mucus exhibits a different protein composition from that of the respiratory mucus^18,21^. Comparison of the top 10 most abundant proteins in the OCLF and respiratory mucus revealed 7 proteins common to both sample types (Supplementary Table 3), suggesting that these proteins are universally present in airway mucosa. However, other proteins were specific to either the OCLF or respiratory mucus proteome, suggesting that they have specific functions in each mucosal area. LCN15 was detected as an olfactory specific protein and was the second most abundant protein in OCLF, suggesting a specific role in olfactory mucus.

Six of the 10 most abundant olfactory mucus proteins identified by Wolf et al.^20^ overlapped with those in our OCLF proteome, suggesting the validity of our sampling method. We identified LCN15, LCN2, LCN1, and very low levels of alpha-1-microglobulin/bikunin precursor, apolipoprotein D, and retinol-binding protein in our OCLF proteome analysis. Unexpectedly, OBPs were not detected in the OCLF. Proteome analyses of human olfactory mucus by Briand et al.^18^ and Debat et al.^19^ identified OBPs in the olfactory mucus, but did not detect LCN15. In contrast, more recent proteome analyses have detected LCN15 in human olfactory mucus^21^, but not OBPs^20,21^. The reason underlying this discrepancy remains unclear, but several possible explanations exist. First, differences in sample population can contribute to such differences in results. Second, differences may be attributed to the variety of sampling methods for olfactory mucus analysis. Three previous studies used microcatheter insertion in the olfactory cleft^18–20^, and one used surgical sponge insertion^21^, whereas we collected olfactory mucus by the olfactory cleft lavage with 1-mL saline for the proteome analysis. Furthermore, differences in sample processing methods, such as condensation and digestion of proteins, may have affected the results^20^. Finally, protein sequence databases change over time, potentially causing discrepancies. Because LCN15 first appeared in the Swiss-Prot protein sequence database in 2009, it is likely that LCN15 was not included in analyses conducted prior to that year^18,19^.

Both OCLF and OCMS samples showed higher LCN15 concentrations than those in the respiratory mucus. Furthermore, OCLF showed an approximately tenfold higher ratio of LCN15 to total protein than that for OCMS. This result, taken together with the finding that LCN15 immunoreactivity was restricted to the olfactory mucosa in human nasal sections (Fig. 2), suggests that OCLF samples better represent olfactory mucus than OCMS samples. This may be because the human olfactory mucosa is located deep inside the olfactory cleft^25^, and the saline used for irrigation could easily reach the area and collect the olfactory mucus. Irrigation of the olfactory cleft, therefore, represents an easy, reliable, and non-invasive method to collect human olfactory mucus.

Quantitative image analysis showed that the distributions of LCN15 and PGP9.5 immunoreactivity were correlated. This finding suggests a relationship in the distributions of LCN15 and ORNs in the olfactory cleft. On the contrary, the ratio of LCN15-negative glands / total CK18-positive glands did not correlate the area of PGP-9.5 immunoreactivity. The identity of LCN15-negative glands in the olfactory cleft mucosa remains unclear, but one possibility is that they represent degenerated Bowman’s glands, as suggested by a previous animal study^29^. Another possibility is that they are glands in the respiratory mucosa, which are also CK18-positive^26,27^.

The patterns of LCN15 levels between older and younger patients with and without olfactory impairment suggest that LCN15 production exhibits an age-related decline. Previous human histological^25,30,31^ and animal^29,32–34^ studies have demonstrated that the area of the olfactory mucosa decreases with age, and that the olfactory mucosa itself shows age-related degeneration. Lower LCN15 concentration in the olfactory mucus in ≥ 50 years age groups, along with the distribution correlation of LCN15 and PGP9.5 immunoreactivity in the olfactory mucosa, suggests that subclinical degeneration of the olfactory mucosa may occur in the elderly, even in individuals with a normal range of olfactory function.

In vitro luciferase assay demonstrated that all EC50 values in the LCN15 group were within the logEC50 95% CI of the corresponding albumin group, suggesting no statistical significance between LCN15 and albumin groups regarding OR response for any of the tested OR-odorant combination. We also examined the effects of LCN15 on bacterial growth. Although the result was preliminary due to the limited availability of recombinant LCN15 protein and bacterial species, LCN15 showed mild bacteriostatic effects on *E. coli* in the iron-limiting culture medium. Further studies will be necessary to confirm these preliminary results.

In rodents, LCNs are expressed in vomeronasal organs^10,12^. LCNs/OBPs are involved in the perception of pheromones and play specific roles. Although the presence and function of residual vomeronasal glands in human are under debate^35–37^, findings in other mammals suggest that LCN15 may have a function in the vomeronasal system. Further study on the expression of LCN15 in residual vomeronasal organs is necessary.

We recognize several limitations in our study. First, our immunohistochemistry results were based only on samples from patients with tumors. Therefore, the results may not be representative of all healthy individuals. Furthermore, location of the LCN15-positive glands within the olfactory mucosa could not be identified accurately via histological analysis because of the variability of surgical tissue resection in each patient and because the direction of the tissue in the archival paraffin block was not uniform. Third, as described above, our bacteriostatic assay was preliminary; because of the limited availability of the recombinant LCN15 protein, we could prepare only a small number of cultures for each time point (Supplementary Fig. 9), and a dose-dependent response analysis was not performed. Furthermore, because of a technical limitation, we used *Escherichia coli*, which is not a resident bacterial species in the nasal mucosa. In the future, we aim to test the bacteriostatic effects on *Streptococcus* bacteria or *Haemophilus influenza*, which are prevalent in the nasal mucosa.

In conclusion, we identified LCN15 as an abundant component of the olfactory mucus, produced in the Bowman's glands of the olfactory mucosa. The concentration of LCN15 in OCLF was lower in the normal olfaction participants (≥ 50 years) and also tended to be lower in the patients with idiopathic olfactory impairment (≥ 50 years) than in normal olfaction participants (< 50 years). Furthermore, LCN15 immunoreactivity along the olfactory cleft mucosa was significantly correlated with the area of neuron-specific PGP9.5 immunoreactivity, suggesting that LCN15 is produced in non-degenerated areas of the olfactory neuroepithelium. Although further investigation is necessary to address the function of LCN15 in the olfactory mucus, our results suggest that LCN15 may serve as a biomarker for Bowman’s gland activity.

## Supplementary Information

Supplementary Information.

## Data Availability

The data that support the findings of this study are available from the corresponding author on reasonable request.
