# Supplementary material for: Lipocalin 15 in the olfactory mucus is a biomarker for Bowman’s gland activity
Source: Sci Rep. 2022 Jun 24;12:9984. doi: 10.1038/s41598-022-13464-y (PMC9232505; doi:10.1038/s41598-022-13464-y)

Supplementary Information

for

Lipocalin 15 in the olfactory mucus is a biomarker for Bowman's gland activity

Chiori Ijichi[1,*], Kenji Kondo[2,*], Masayoshi Kobayashi[3], Ayaka Shirasawa[4], Kazutaka Shimbo[4], Kunio Nakata[4], Yutaka Maruyama[1], Yusuke Ihara[1], Yayoi Kawato[1], Teruhisa Mannen[4], Rie Takeshita[4], Yoshimi Kikuchi[4], Yuki Saito[2], Tatsuya Yamasoba[2]

[1]Technology & Solution Development Center, Institute of Food Science and Technologies, Food Products Division, Ajinomoto Co., Inc., Kawasaki, Japan

[2]Department of Otorhinolaryngology-Head and Neck Surgery, Graduate School of Medicine and Faculty of Medicine, The University of Tokyo, Tokyo, Japan

[3]Department of Otorhinolaryngology-Head and Neck Surgery, Mie University Graduate School of Medicine, Tsu, Mie, Japan

[4]Research Institute for Bioscience Products & Fine Chemicals, Ajinomoto Co., Inc., Kawasaki, Japan

Supplementary Table 1.

Characteristics of archival olfactory cleft tissue samples

Patient	Age	Sex	Pathology
1	69	F	Olfactory neuroblastoma
2	68	F	Olfactory neuroblastoma
3	65	M	Olfactory neuroblastoma
4	62	M	Olfactory neuroblastoma
5	26	M	Adenocarcinoma
6	67	F	Squamous cell carcinoma
7	57	M	Squamous cell carcinoma
8	48	F	Adenosquamous carcinoma
9	80	M	Myoepithelial carcinoma
10	71	M	Squamous cell carcinoma

Supplementary Table 2

Top 10 most concentrated proteins in the olfactory cleft lavage fluid (OCLF) and respiratory mucus, as determined by proteome analysis

OCLF

Rank	Protein	OCLF	Respiratory mucus
1	Polymeric immunoglobulin receptor	407±72	148±27
2	Lipocalin-15	339±163	12±4
3	Immunoglobulin heavy constant alpha 1	304±74	155±56
4	Serum albumin	298±42	444±96
5	Lactotransferrin	255±64	490±189
6	Lysozyme C	251±72	135±43
7	Fibronectin	232±3	1±1
8	BPI fold-containing family B member 1	172±68	165±105
9	Prolactin-inducible protein	171±34	284±24
10	Transcobalamin-1	156±18	41±4

Respiratory mucus

Rank	Protein	Respiratory mucus	OCLF
1	Lactotransferrin	490±189	255±72
2	Serum albumin	444±96	298±163
3	Prolactin-inducible protein	284±24	171±42
4	BPI fold-containing family B member 1	165±105	172±64
5	Immunoglobulin heavy constant alpha 1	155±56	304±72
6	Polymeric immunoglobulin receptor	148±27	407±3
7	Lysozyme C	135±43	251±68
8	Zymogen granule protein 16 homolog B	118±26	41±26
9	Antileukoproteinase	103±25	151±25
10	Serotransferrin	97±19	66±19

*Quantitative scores in this Table were calculated from spectral counting by using Scaffold software.

Supplementary Table 3

Half maximal effective concentration (EC50) of albumin and LCN15 supplementation on olfactory receptor activity by odorant binding.

OR-odorant combination		LCN15	Albumin	PBS
OR10G7-eugenol	LogEC50	−6.493	−6.535	−6.647
	LogEC50_95% CI	−7.356 to −6.193	−6.697 to −6.413	−7.018 to −6.439
OR5AN1-muscone	LogEC50	−6.206	−6.063	−6.069
	LogEC50_95% CI	−6.495 to −6.012	−6.232 to −5.927	−6.166 to −5.984
OR51E1-isovaleric acid	LogEC50	−4.976	−4.942	−5.161
	LogEC50_95% CI	−5.223 to −4.718	−5.103 to −4.774	−5.251 to −5.070
OR2W1-benzylacetate	LogEC50	−4.695	−4.546	−4.53
	LogEC50_95% CI	−5.134 to −4.119	−4.989 to −3.942	−4.862 to −4.111
OR10G4-ethylvanillin	LogEC50	−5.228	−5.185	−5.315
	LogEC50_95% CI	−5.534 to −4.914	−5.295 to −5.073	−5.501 to −5.131
OR1A1-(-)carvone	LogEC50	−5.926	−5.864	−5.944
	LogEC50_95% CI	−6.016 to −5.845	−5.932 to −5.801	−6.105 to −5.809

CI: confidence interval

Supplementary Materials and Methods

Proteomics

Each mucus sample containing 20 μg of total protein in 100 mM ammonium bicarbonate (FUJIFILM Wako Pure Chemical Corporation, Osaka, Japan) buffer was reduced with 10 μL of 60 mM dithiothreitol (Thermo Fisher Scientific). After incubation at 55 °C for 15 min, 15 μL of 90 mM iodoacetic acid (FUJIFILM Wako Pure Chemical Corporation) was added at 23 °C for 30 min in the dark to alkylate free thiols. One hundred microliters of 100 mM ammonium bicarbonate buffer and 15 μL of 50 ng/μL trypsin (Sigma-Aldrich) were added to the sample and incubated at 37 °C for 18 h. After the digestion, 10 μL of 10 % trifluoroacetic acid (FUJIFILM Wako Pure Chemical Corporation) was added to terminate the digestion. The resulting digestion samples were dilute to twice their volume with 0.1 % formic acid in 2 % acetonitrile aqueous solution for nano liquid chromatography tandem mass spectrometry (LC-MS/MS) analysis. The resulting peptide mixture was analyzed on Orbitrap Fusion wETD system (Thermo Fischer Scientific, San Jose, CA) equipped with Dream Spray for Thermo nano ion source (AMR, Inc., Tokyo, Japan) and Easy-nLC 1000 (Thermo Fischer Scientific). We used an Acclaim PepMap® 100 (75 μm × 2 cm, Thermo Fischer Scientific) for the trap column and an ESI-column (75 μm × 12.5 cm, 3 μm, NTCC-360/75-3-125, Nikkyo Technos, Tokyo, Japan) for the analytical column. The chromatographic procedure comprised a 0.5 min hold at 2 % solvent B (0.1 % formic acid in acetonitrile, Thermo Fischer Scientific) and 78 min linear gradient from 2 % to 35 % solvent B. The next wash step was performed as 12 min linear gradient from 35 % to 75 % solvent B followed by a 9.5 min hold at 75 % solvent B. The solvent A consisted of 0.1 % formic acid (Thermo Fischer Scientific). Mass spectrometry analysis was carried out in a data dependent acquisition (DDA) mode with full scans (*m/z* 350–2,000) acquired at a mass resolution of 120,000. The spray voltage and ion transfer tube

temperature were set to 1600 V and 275 °C, respectively. Among detected ions, charge states other than 2–4 were filtered out and the 10 most intense ions were selected for MS/MS analysis. The tandem mass spectra were produced by collision induced dissociation (CID) method. An AGC target ion number for MS1 and MS2 were set to 4.0e5 and 1.0e4, respectively. The maximum injection time for both MS1 and MS2 was set to 50 ms. For the dynamic exclusion, the duration was set to 15 s. All resulting MS/MS samples were analyzed against the Swiss-Prot Human database (2017.7, 20215 entries) using the Mascot (version 2.6.0, Matrix Science Inc, London, UK) search engine. Trypsin was selected for digestion. The mass tolerance of precursor ions (MS1) and fragment ions (MS2) was set to 10 ppm and 0.8 Da, respectively. Cysteine carboxymethylation was specified as a fixed modification, and methionine oxidation as a variable modification. Scaffold (version 4.8.3, Proteome Software, Inc, Portland, OR) was used to integrate the proteomics data to compare the total spectrum counts. The thresholds for the protein false discovery rate (FDR), peptide FDR, and number of peptides per protein were set to 1.0 %, 0.1 %, and 2, respectively.

Preparation of hLCN15 protein

The primary amino acid sequence of mature hLCN15 (see below) was obtained from UniProtKB (accession number: Q6UWW0):

EVLLQPDFNAEKFSGLWYVVSMASDCRVFLGKKDHLSMSTRAIRPTEEGGLHVHME
FPGADGCNQVDAEYLKVGSEGHFRVPALGYLDVRIVDTDYSSFAVLYIYKELEGALS
TMVQLYSRTQDVSPQALKSFQDFYPTLGLPKDMMVMLPQSDACNPESKEAP

The DNA fragment was chemically synthesized (Eurofins Genomics, Tokyo, Japan) and subcloned into pET16b (Merck, Darmstadt, Germany). Recombinant hLCN15 with a 6X His-tag, followed by a Factor Xa recognition site in the *N*-terminus, was transformed into

Escherichia coli BL21 (DE3) using the heat-shock method. The transformants were cultured in Magicmedia™ (Thermo Fisher Scientific, Waltham, MA) at 37 °C for 16 h, followed by fermentation at 20 °C for 20 h. Harvested cells were suspended in 50 mM Tris-HCl solution (pH 9.0) containing 1 M NaCl, 2 mM $MgCl_2$, 2 mM $CaCl_2$, and 10 % glycerol, and disrupted by sonication. Cell debris was excluded by centrifugation, and the supernatant was purified using HIS-Select® Nickel Affinity Gel (Sigma Aldrich, St. Louis, MO, USA) according to the manufacturer's instructions. Elution buffer containing hLCN15 was exchanged with 50 mM Tris-HCl (pH 8.0), 100 mM NaCl, 5 mM $CaCl_2$, and 1 mM dithiothreitol (DTT), and then concentrated using Amicon Ultra MWCO10k (Merck Millipore, Billerica, MA). The hLCN15 solution was incubated with Factor Xa (Merck Millipore) at 37 °C for 120 h to cleave the *N*-terminal His-tag. The cleaved His-tag was eliminated by Ni-affinity gel to obtain purified hLCN15.

The purity of the recombinant hLCN15 was confirmed by SDS-PAGE, LC/MS intact protein mass analysis, and LC/MS/MS peptide mapping analysis. For LC/MS intact protein mass analysis, a sample solution was prepared by adding 4 μL of 8 M urea/100 mM ammonium bicarbonate to 6 μL of 500 mg/mL hLCN15 and diluted 5-fold with 0.1% formic acid and 9% acetonitrile. The sample solution was analyzed using an ACQUITY UPLC (Waters, Milford, MA) coupled to an Orbitrap Fusion wETD (Thermo Fisher Scientific) mass spectrometer. The resulting LC/MS data were analyzed using BioPharma Finder 3.2 (Thermo Fischer Scientific). For the LC/MS/MS peptide mapping analysis, the same analytical conditions were used as described in 'Proteomics' above, and the recombinant human LCN15 sequence was used for sequence annotation.

Measurement of olfactory receptor activity

The luciferase reporter gene assay was performed as previously described [8] using the Dual-Glo Luciferase Assay System (Promega).

To examine the effect of LCN15 on olfactory receptor activity, seven pairs of ORs and their agonists were selected (OR10G7 and eugenol, OR5AN1 and muscone, OR51E1 and isovaleriac acid, OR2W1 and benzylacetate, OR5P3 and cumarine, OR10G4 and ethyl vanillin, OR1A1 and (-)-carvone.) HEK293T cells were transfected with 12.5 ng of each tagged OR vector, 2.5 ng CRE/luc2P, 1.25 ng TK/Rluc, 2.5 ng RTP1S-pcDNA3.1 vector, 1.25 ng Ric8B-pcDNA3.1 vector, and 1.25 ng Golf-pcDNA3.1, using 0.0425 μL Lipofectamine 2000 (Invitrogen, Carlsbad, CA, USA) per well. At 24 h after transfection, the medium was replaced with CD293 (Invitrogen) containing 1/10000 v/v GlutaMAX (Thermo Fisher Scientific, Waltham, MA, USA) and the cells were stimulated by either of the following odorants for 4 h at 37 °C and 5 % CO_2:

1. An odorant with 3–100 μM and LCN15 (25 μM) in PBS
2. An odorant with 3–100 μM with albumin (25 μM) in PBS
3. An odorant with 3–100 μM in PBS

Luminescence was detected using the FDSS7000EX functional drug screening system (Hamamatsu Photonics, Hamamatsu, Japan). Fold-increases were calculated as Luc/hRLuc (Luc [luminescence of firefly luciferase] divided by hRLuc [luminescence of *Renilla* luciferase]) stimulated with an odorant divided by Luc/hRLuc upon no stimulation.

Bacterial growth inhibition tests

The tests were performed in collaboration with the OP Bio Factory Co., Ltd. (Okinawa, Japan). To test the bacteriostatic activity of LCN15, the *E. coli* strain NBRC102203 was grown in 10 mL M9 minimal medium in culture tubes at 37 °C and 200 rpm for 18 h. The culture was then

diluted to a concentration of 1×10^2 cells/mL with medium and transferred to 96-well dishes (Greiner, Wiesbaden, Germany) at 40 μL/well. The plates were incubated at 37 °C and 800 rpm. LCN15 was added at a concentration of 5 μM. The optical density at 600 nm (OD600) was measured after 1, 4, 24, 28, 30, and 48 h.

Supplementary Figure Legends

Supplementary Fig 1.

Head position of the subject during olfactory cleft lavage. Participants took the prone position, with knees bent, and the top of the forehead on the floor.

Supplementary Fig. 2.

Purity analysis of recombinant human LCN15. **a:** SDS-PAGE results of the recombinant protein revealed a band at approximately 18 kDa, corresponding to LCN15. **b:** Intact protein mass spectrometry analysis of recombinant LCN15. There are two peaks; the first peak at 18319 Da corresponds to human LCN15. The identity of molecule at the second peak (18362 Da) was not determined, but its molecular weight suggests an acetylated form of the recombinant LCN15.

Supplementary Fig 3.

Magnified views of the human olfactory cleft mucosa stained with rabbit anti-PGP9.5 antibody (**a** and **d**), rabbit anti cytokeratin 18 antibody (**b** and **e**), and rabbit anti-LCN15 antibody (**c** and **f**). Images X and Y correspond to the microscopic fields X and Y indicated by red rectangles in **Fig. 2. a:** PGP9.5-positive olfactory neuroepithelium (black arrowheads) in the superior part of the olfactory cleft mucosa. **d:** in the lower end of the olfactory cleft, PGP9.5 immunoreactivity is not observed (right half of the image; in the left half epithelium has been lost during harvest). **b:** In the area corresponding to **a,** CK immunoreactivity is observed both in the epithelium and submucosal glands. **e:** CK immunoreactivity in the epithelium and submucosal glands. As shown in **a,** epithelium in the left half of the image is missing due to

the artifact. **c:** In comparison with **b,** LCN15 immunoreactivity is observed in a part of the submucosal glands in the superior olfactory cleft. **f:** There is no LCN15 immunoreactivity in the inferior end of the cleft mucosa. Scale bar in F (also applies to **a**–**e**) = 500 µm

Supplementary Figure 4.

Representative photomicrograph of olfactory cleft mucosa, double-stained with anti-PGP9.5 and anti-LCN15 antibodies. After antigen retrieval, the section was first incubated with rabbit anti-LCN15 antibody. The immunoreactivity was visualized using HRP-conjugated anti-rabbit IgG secondary antibody and DAB reaction (blown color, black arrows). The section was then placed in a citrate buffer solution (pH 6.0) and autoclaved at 121 °C for 5 min to deplete antigenicity of the anti-LCN15 primary antibody to the secondary antibody and to inactivate enzymatic activity of HRP binding to anti-rabbit IgG antibody. The section was then incubated with rabbit anti-PGP9.5 antibody. The immunoreactivity was visualized using HRP-conjugated anti-rabbit IgG antibody again and Vector SG reaction (Gray color, black arrowheads). Scale bar = 100 µm.

Supplementary Fig. 5.

Correlation of LCN15- and PGP9.5-immunoreactive areas in olfactory cleft mucosal zones. The olfactory cleft mucosa of each sample was divided into four zones (dorsomedial, dorsolateral, ventromedial, and ventrolateral), and rectangular areas of each were analyzed (Fig. 4). We then compared the areas of PGP9.5- and LCN15-immunoreactivity in each rectangle. Coefficients of determination between the two immunoreactive areas tended to be higher in the ventromedial (R^2 = 0.3985) and ventrolateral (R^2 = 0.2922) areas than in the dorsomedial (R^2 = 0.2314) and ventrolateral (R^2 = 0.1556) areas.

Supplementary Fig. 6.

Correlation between the distribution of LCN15-negative/CK18-positive glands and PGP9.5-immunoreactive area in the olfactory cleft mucosal zones. The olfactory cleft mucosa was divided into four zones, and the number of acini of LCN15- and CK18-positive glands were manually counted in each field. We calculated the ratio of the number of LCN15-negative glands/CK18-positive glands in each rectangle, then compared it with the area of PGP9.5 immunoreactivity. Coefficient of determinations between the parameters was less than 0.2 in each of four zones, indicating there was no statistical correlation.

Supplementary Fig. 7.

Comparison of LCN15 concentration in OCLF of patients with normal olfaction and with idiopathic olfactory impairment. Each group was further divided into two subgroups by age (<50 years and ≥50 years). Each symbol represents the value of each individual. IOI: idiopathic olfactory dysfunction. *P* values (Tukey's post-hoc test) between the subgroups are presented on the top of the graph.

Supplementary Fig. 8.

Effects of LCN15 supplementation on OR activity by odorant binding. The following six pairs of ORs and their agonists were selected and used in the luciferase assay: OR10G-eugenol, OR5AN1-muscon, OR51E1-isovaleric acid, OR2W1-benzylacetate, OR10G4-ethylvanillin, and OR1A1-carvone. The dose-dependent analysis was performed with 3–100 μM of each odorant supplemented with LCN15 (25 μM), bovine serum albumin (25 μM), or PBS. In each graph, the X-axis indicates the concentration of the odorant in log scale, and the Y-axis indicates the fold increase of the olfactory receptor (OR) response in log scale. Dose response curves

show each OR-odorant response under each medium condition. As shown in Supplementary Table 3, in two OR-odorant combinations (ORAN1-muscone and OR51E1-isovaleic acid), the EC50 of LCN15 and albumin groups were out of the logEC50 95% CI of the control group, suggesting that LCN15 and albumin may affect odorant perception in these combinations. However, all EC50 values in the LCN15 group were within the logEC50 95% CI of the albumin group, suggesting no statistical significance between LCN15 and albumin groups in any OR-odorant conbination.

Supplementary Fig. 9.

Bacteriostatic effects of LCN15 on the growth of *E. coli. E. coli* strain NBRC102203 was cultured in M9 minimal medium at a dilution of 1×10^2 cells/mL medium in 96-well dishes. LCN15 was added at 5 μM. The optical density at 600 nm (OD600) was measured after 1, 4, 24, 28, 30, and 48 h. The number of cultures for the control was n = 2 (1, 4, 24, 28, 30, and 48 h), and that for the LCN15 group was n = 2 (1, 4 h) and n = 1 (24, 28, 30, and 48 h). After the addition of LCN15 at a concentration of 5 μM, approximately 35 % inhibition of growth was observed.

Supplemetary Figure 1

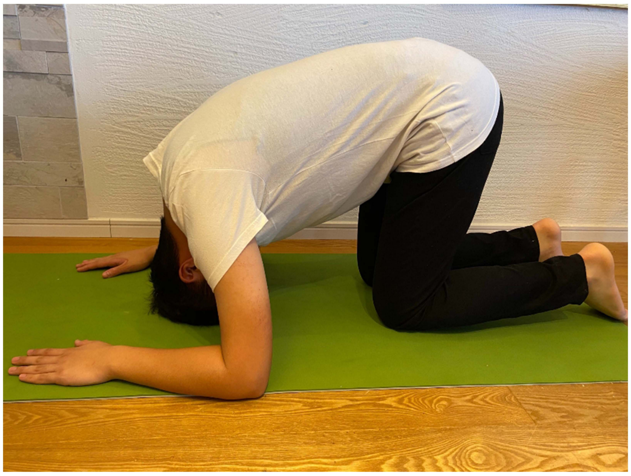

Supplementary Figure 2

a

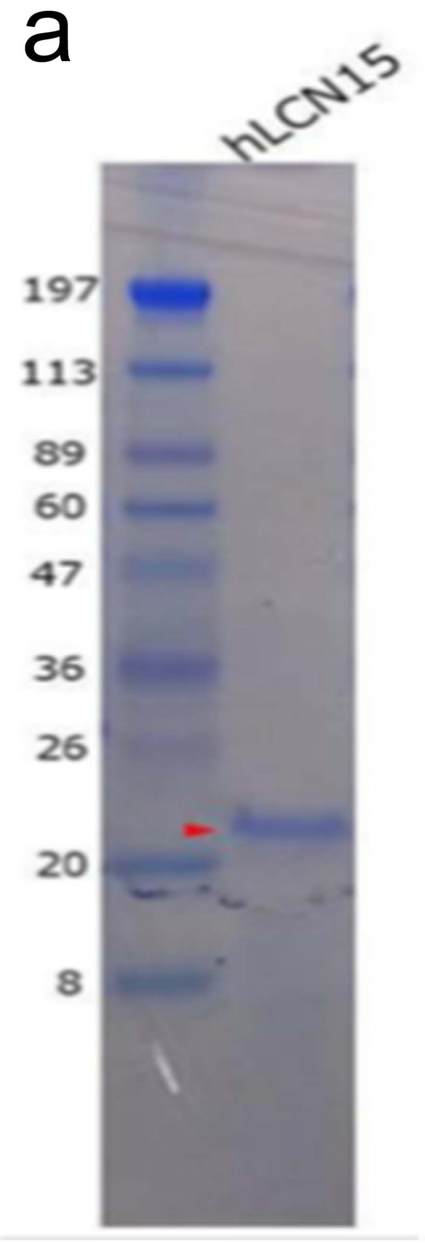

b

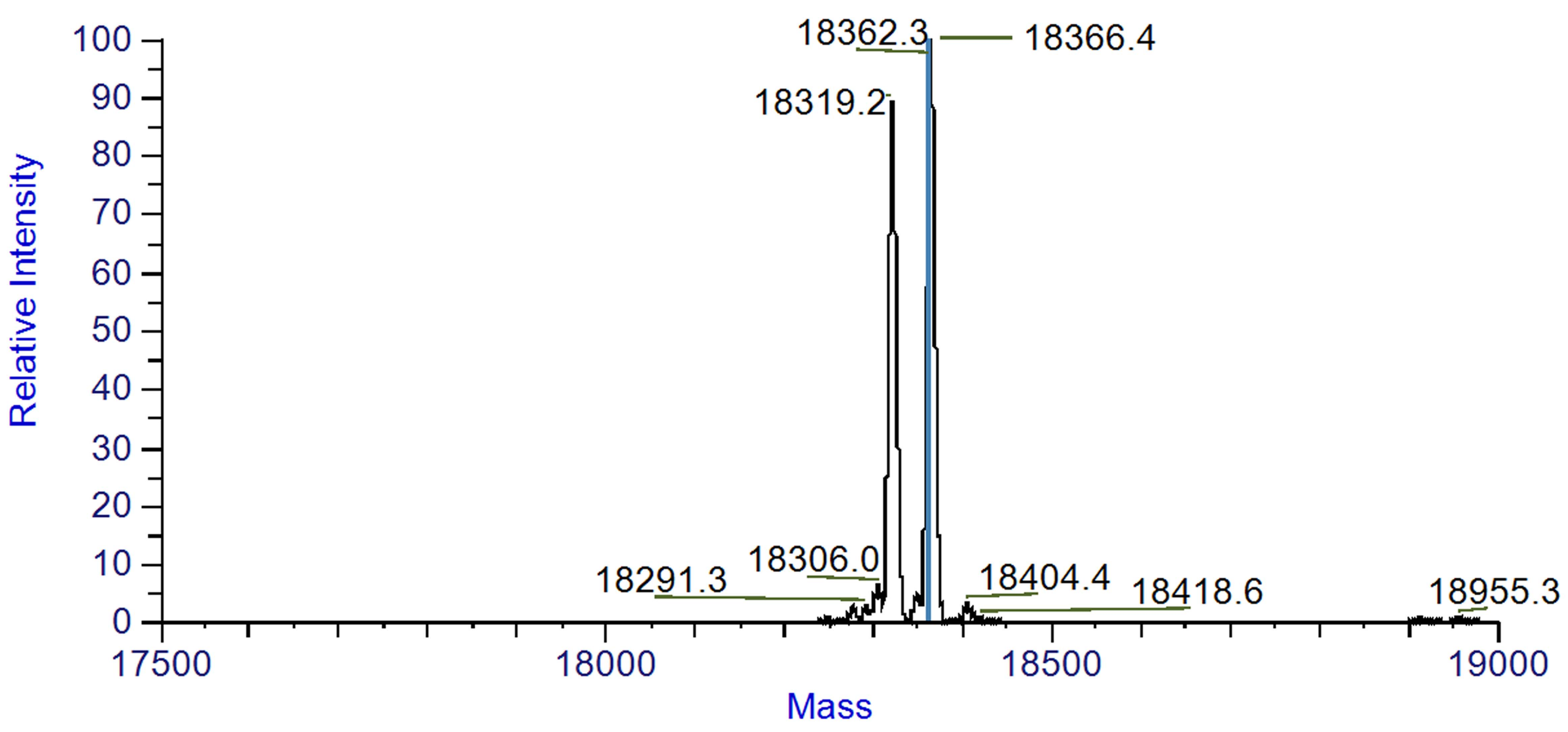

Supplementary Figure 3

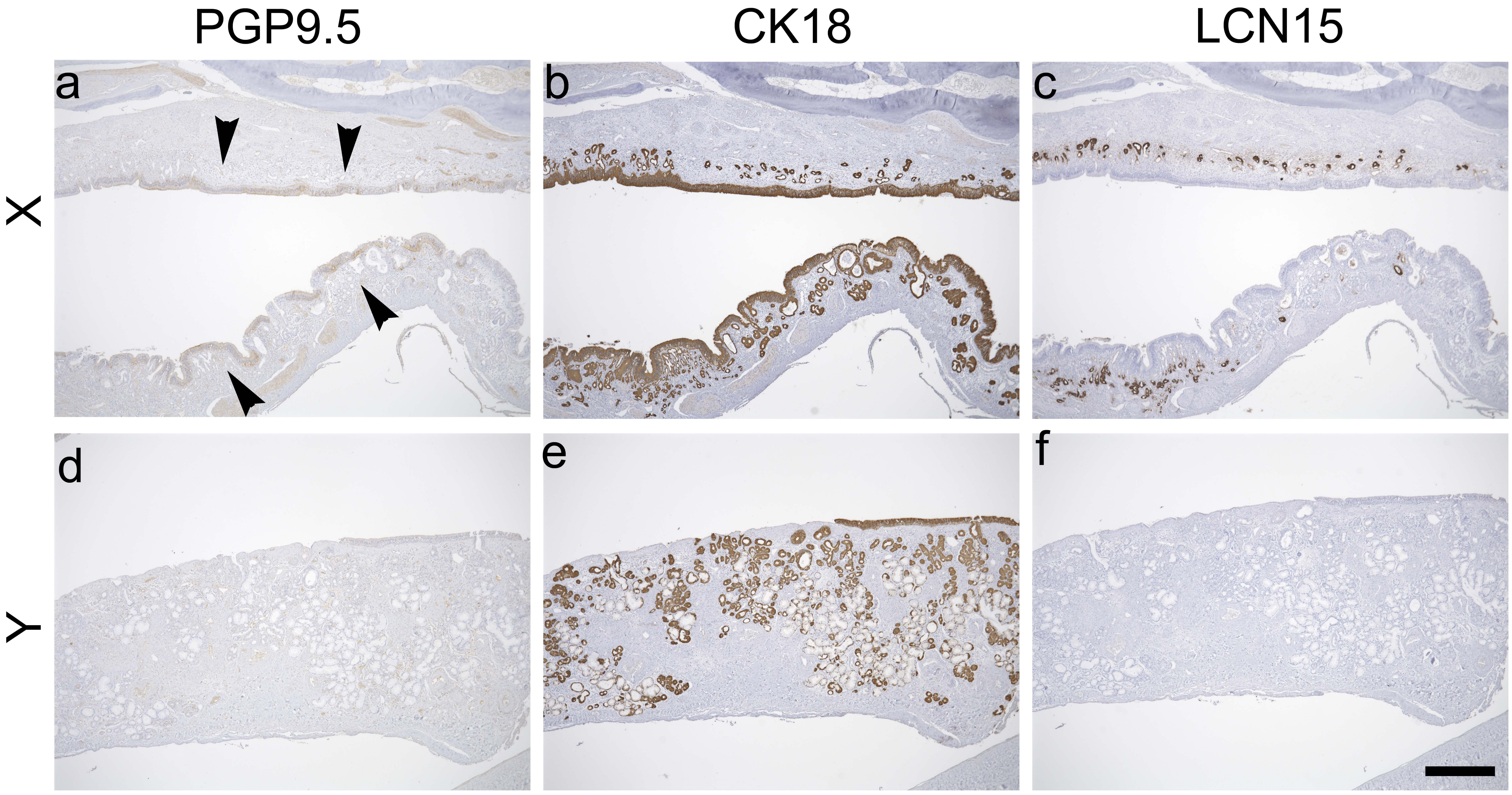

Supplementary Figure 4

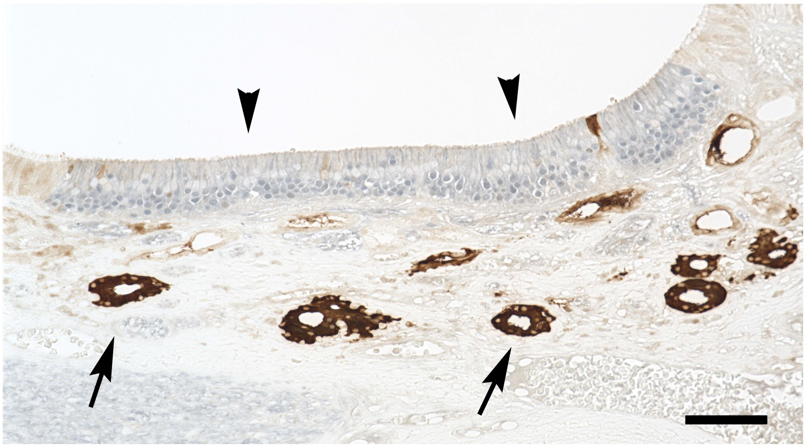

Supplementary Figure 5

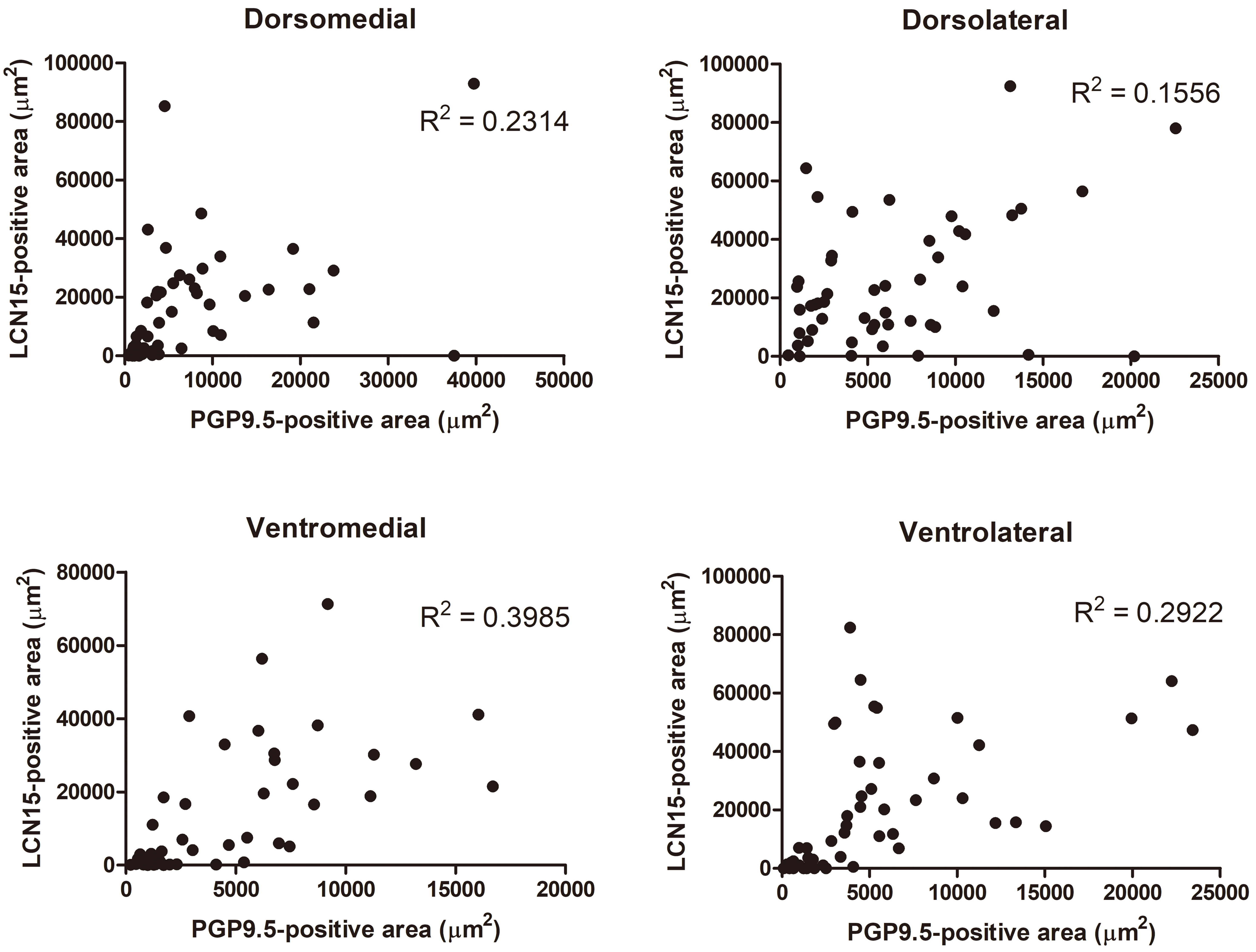

Supplementary Figure 6

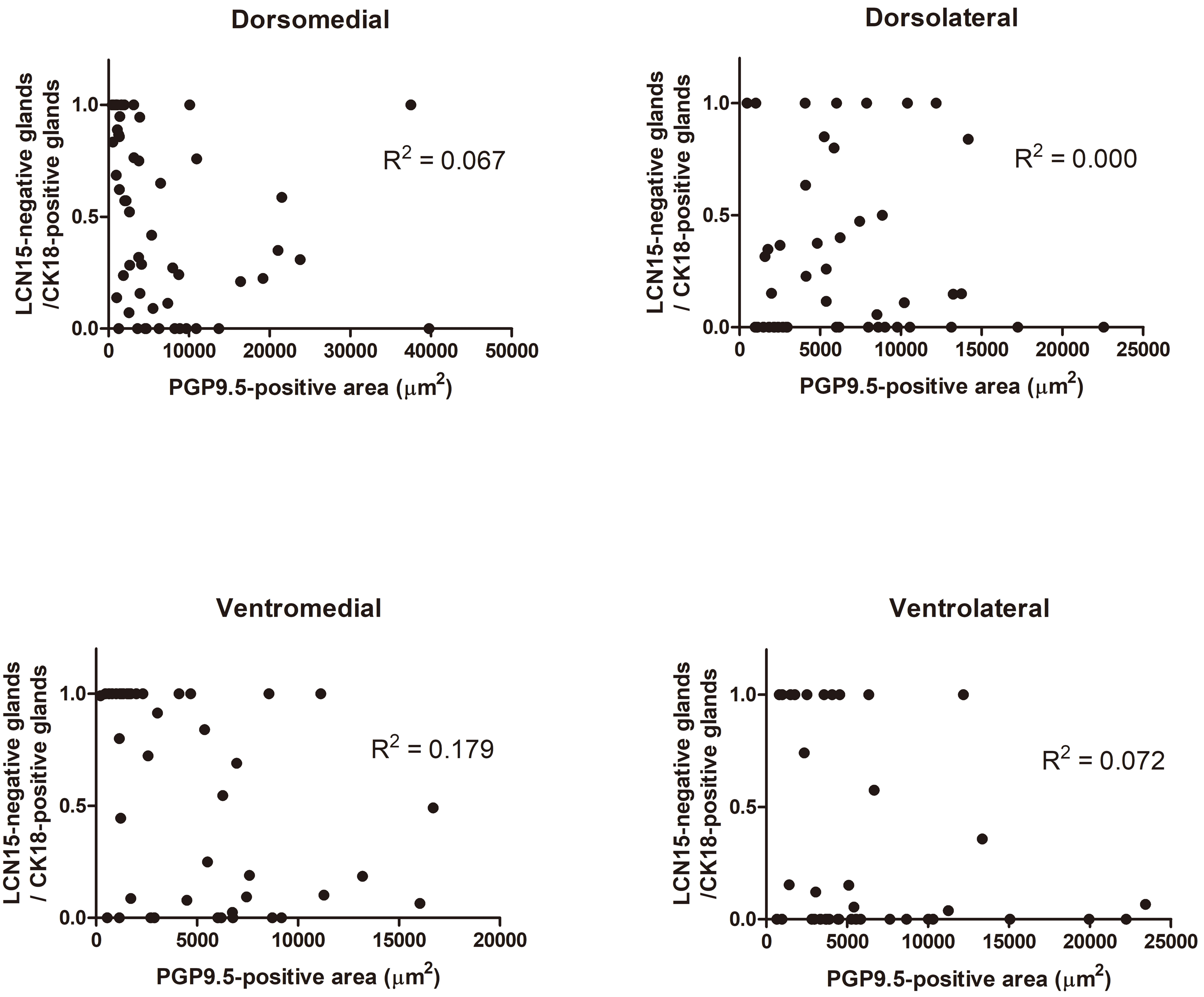

Supplementary Figure 7

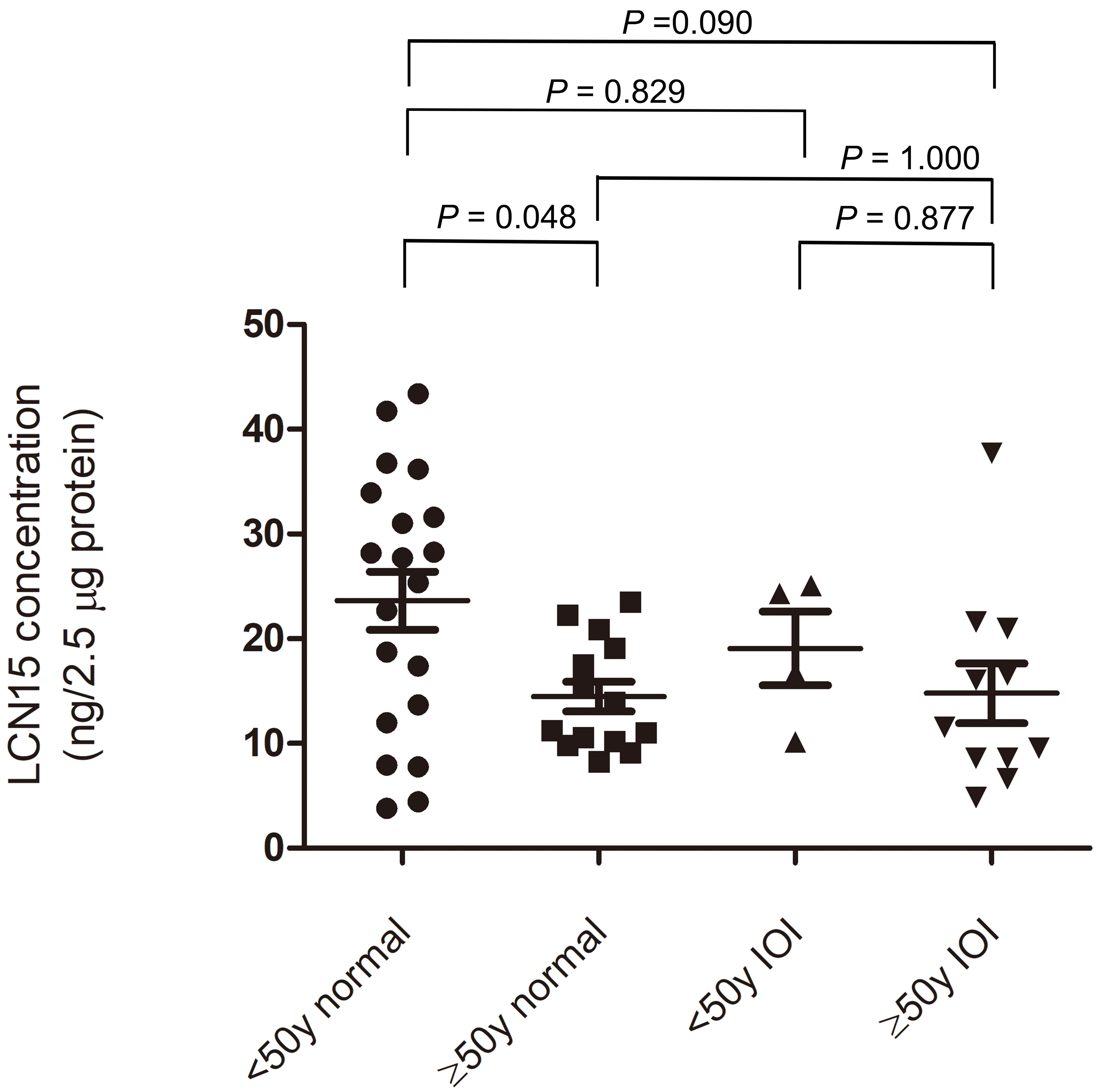

Supplementary Figure 8

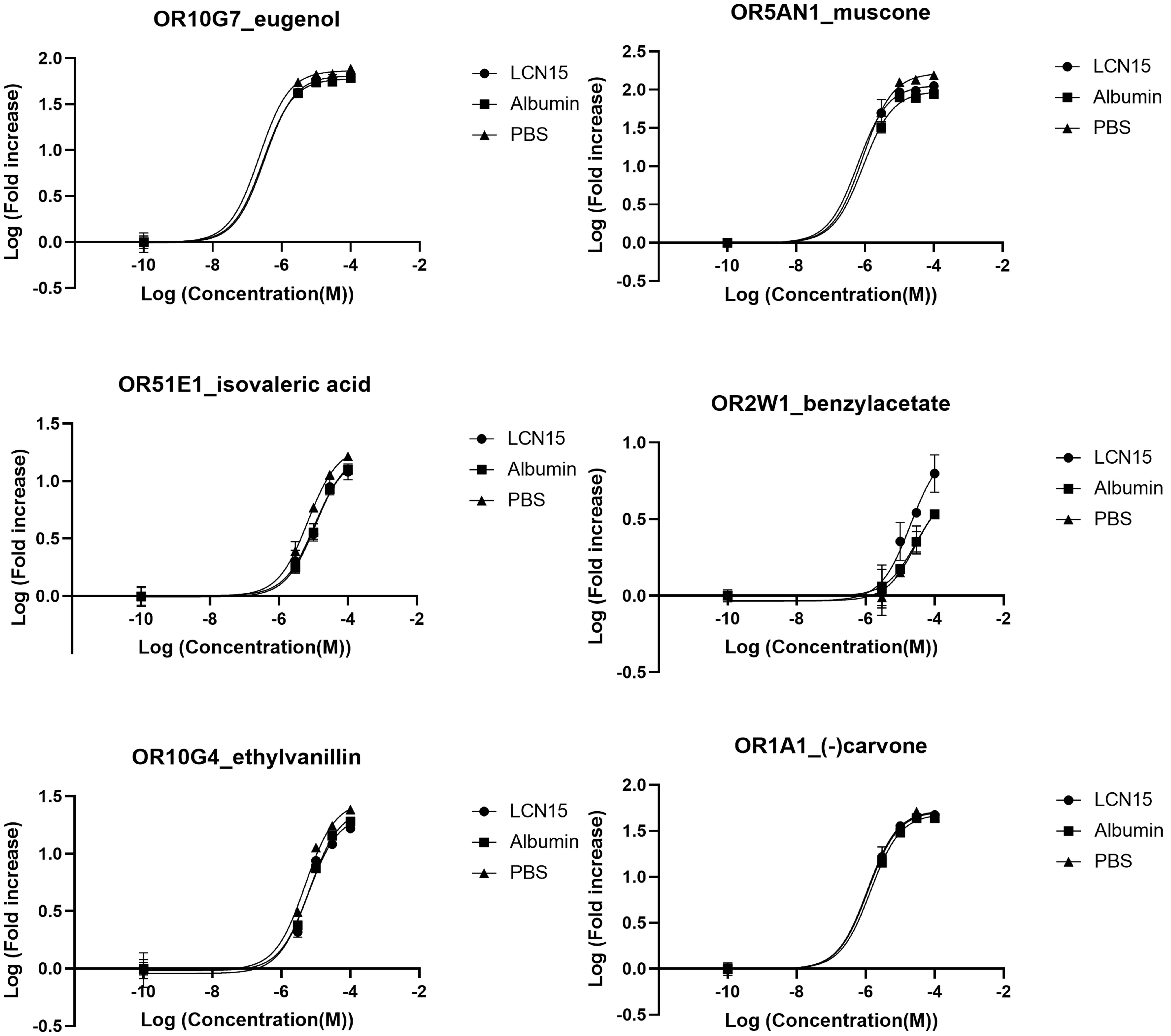

Supplementary Figure 9

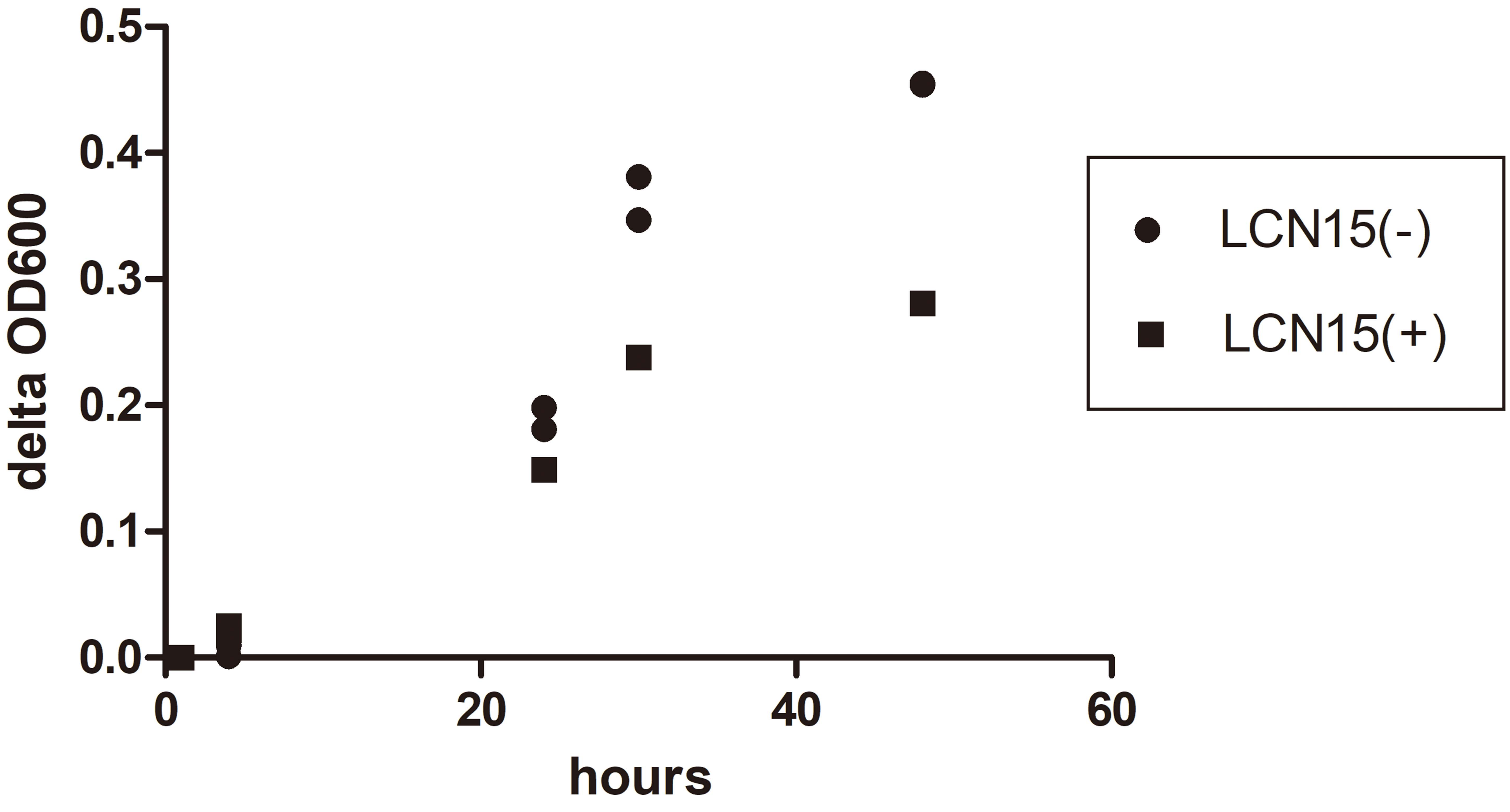

Supplement: Supplementary file 1 — Supplementary Information. [file 41598_2022_13464_MOESM1_ESM.pdf]
